# Thousands of oscillating LncRNAs in the mouse testis

**DOI:** 10.1016/j.csbj.2023.11.046

**Published:** 2023-11-29

**Authors:** Shital Kumar Mishra, Taole Liu, Han Wang

**Affiliations:** a Center for Circadian Clocks, Soochow University, Suzhou 215123, Jiangsu, China; b School of Biology & Basic Medical Sciences, Suzhou Medical College, Soochow University, Suzhou 215123, Jiangsu, China

**Keywords:** Mouse, Testis, Circadian rhythmicity, LncRNAs, LncRNA-encoded peptides, Bioinformatics

## Abstract

The long noncoding RNAs (lncRNAs) are involved in numerous fundamental biological processes, including circadian regulation. Although recent studies have revealed insights into the functions of lncRNAs, how the lncRNAs regulate circadian rhythms still requires a deeper investigation. In this study, we generate two datasets of RNA-seq profiles of the mouse (*Mus musculus*) testis under light-dark (LD) cycle. The first dataset included 18,613 unannotated transcripts measured at 12 time points, each with duplicate samples, under LD conditions; while the second dataset included 21,414 unannotated transcripts measured at six time points, each with three replicates, under desynchronized and control conditions. We identified 5964 testicular lncRNAs in each dataset by BLASTing these transcripts against the known mouse lncRNAs from the NONCODE database. MetaCycle analyses were performed to identify 519, 475, and 494 rhythmically expressed mouse testicular lncRNAs in the 12-time-point dataset, the six-time-point control dataset, and the six-time-point desynchronized dataset, respectively. A comparison of the expression profiles of the lncRNAs under desynchronized and control conditions revealed that 427 rhythmically expressed lncRNAs from the control condition became arrhythmic under the desynchronized condition, suggesting a possible loss of rhythmicity. In contrast, 446 arrhythmic lncRNAs from the control condition became rhythmic under the desynchronized condition, suggesting a possible gain of rhythmicity. Interestingly, 48 lncRNAs were rhythmically expressed under both desynchronized and control conditions. These oscillating lncRNAs were divided into morning lncRNAs, evening lncRNAs, and night lncRNAs based on their time-course expression patterns. We interrogated the promoter regions of these rhythmically expressed mouse testicular lncRNAs to predict their possible regulation by the E-box, D-box, or RORE promoter motifs. GO and KEGG analyses were performed to identify the possible biological functions of these rhythmically expressed mouse testicular lncRNAs. Further, we conducted conservation analyses of the rhythmically expressed mouse testicular lncRNAs with lncRNAs from humans, rats, and zebrafish, and uncovered three mouse testicular lncRNAs conserved across these four species. Finally, we computationally predicted the conserved lncRNA-encoded peptides and their 3D structures from each of the four species. Taken together, our study revealed thousands of rhythmically expressed lncRNAs in the mouse testis, setting the stage for further computational and experimental validations.

## Graphical Abstract

**Figure:**
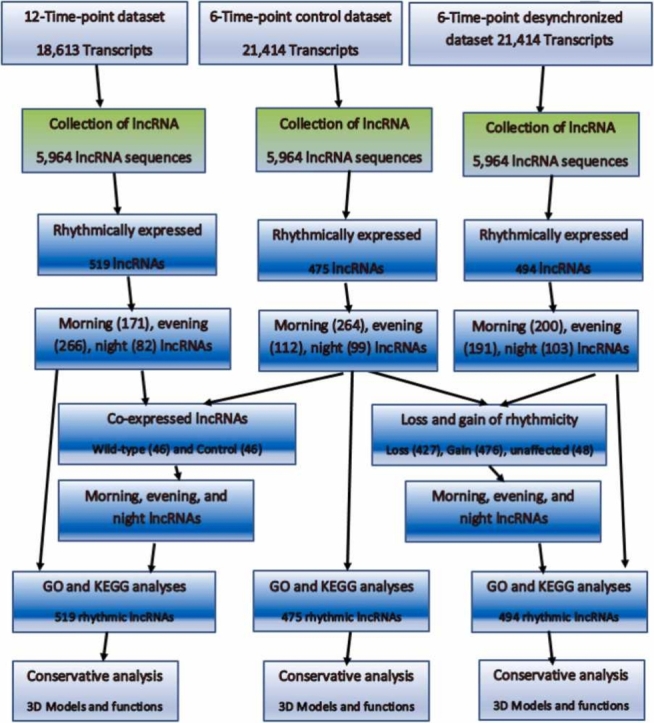

## 1 Introduction

The circadian clock regulates numerous fundamental life processes, such as the coordination of behavior, body temperature, sleep-wake rhythms, and blood pressure [1], [2], [3]. The master clock located in the suprachiasmatic nucleus (SCN) regulates the peripheral organ- and tissue-specific circadian rhythms [4]. Disrupted circadian rhythms are closely associated with the pathogenesis of various diseases, including sleeplessness, immune-allergic diseases, inflammatory bowel disease, and cancers [5], [6]. The circadian rhythms have been studied by employing different model organisms, such as fruit flies (*Drosophila melanogaster*) [7], zebrafish (*Danio rerio*) [8], [9], [10], [11], and mice (*Mus musculus*) [11]. The fruit fly is a suitable organism for studying the circadian clocks in insects [7], and the numerous circadian clock genes were first identified in fruit flies. Further, fruit flies enable convenient genetic manipulations and controlled breeding [12]. The zebrafish is ideal for investigating vertebrates’ circadian rhythms [8], [9], [10], [11]. Since mutational and transgenic zebrafish lines can be generated with ease, zebrafish have been employed to study the onset of circadian rhythmicity, the effects of the light-dark cycle, and locomotor activities. The mice are widely used for studying circadian rhythmicity due to their manageable size, the convenience of genetic manipulations, and their cage environment being suitable for monitoring locomotor activities [11].

Although mice have been reported to share circadian patterns with other organisms, such as zebrafish and humans [13], [14], [15], there are only a few studies investigating circadian rhythmicity in the mouse testis [16]. In fact, a previous study found no circadian rhythms in the mouse testis [17]. The testis is an important organ responsible for regulating reproductive functions, such as producing and storing sperms, and producing testosterone, the primary male sex hormone [18]. However, the evidence for circadian rhythms in the testis remains controversial, including some studies suggesting the testis harbors the circadian rhythms [19], but others arguing against the presence of circadian rhythms in the testis [17]. These studies inspired us to investigate the regulation of circadian rhythms in the mouse testis.

The lncRNAs are classified as over 200-nucleotide-long noncoding RNAs [20]. Despite lncRNAs lacking protein-coding abilities, previous studies revealed that lncRNAs regulate a diverse set of critical life processes, such as gene transcription, cell fate regulation [21], [22], and circadian rhythms [23], [24]. For example, the study validated LINC01018, an obesity-associated lncRNA in humans [25]. We previously identified hundreds of circadianly expressed lncRNAs in zebrafish larvae [26].

Intriguingly, numerous lncRNAs harbor small open reading frames (smORFs) that encode approximately 100 amino-acid-long micropeptides [27], [28], which differ from over 400 amino-acid-long functional proteins encoded by the mRNAs [29]. LncRNAs-encoded micropeptides have been implicated in regulating a variety of life processes, including transcription and regulation of mRNAs [30]. For example, our recent study computationally predicted hundreds of coding lncRNAs in zebrafish [31]. Muscle performance is regulated by myoregulin, a skeletal muscle-specific conserved lncRNA-encoded micropeptide [32]. The lncRNA MIR155HG encoded the micropeptide MIR155HG that regulates autoimmune inflammation [27]. LEMP, a lncRNA MyolncR4-encoded micropeptide, was found to be involved in the regulation of muscle development in mice [30]. A recent study of brain cancer found micropeptides encoded by the smORF of lncRNA DLEU1 [33]. Microprotein FORCP encoded by the smORF of lncRNA LINC00675 is involved in the tumorigenesis [34]. Knockouts of testis-specific lncRNAs have shown the complete loss of fertility in *Drosophila* [35]. Mouse testis-specific lncRNA-Tcam1 regulates immune-related germ cells [36]. Another recent study uncovered polypeptides Kastor and Polluks encoded by mouse locus (Gm9999), previously annotated as mouse testis-specific lncRNAs [37]. Profiling of mouse testicular lncRNAs revealed the expression of numerous lncRNAs in F9, GC-1, and GC-2, all three being germ cell-related cell lines. Further, testis-specific lncRNAs 1700108J01Rik and 1700101O22Rik are expressed in the mouse testicular germ cells [38].

Despite these insights into the functions of lncRNAs and their encoded peptides, their roles in regulating circadian rhythms remain far from complete. In particular, how the circadian clock orchestrates the functions of testicular lncRNAs requires further investigation. We have previously investigated rhythmically expressed lncRNAs in the zebrafish pineal gland, testis, and larvae [9], [31]. In this study, we built upon our previous studies by investigating rhythmically expressed lncRNAs in the mouse testis.

Here, we generated the two datasets of RNA-seq-based time-course profiles of the mouse testis (Fig. S1). The first dataset contains 18,613 unannotated transcripts from the mouse testis (Supplementary Table 1), which was generated from 12 time points for two consecutive days under light-dark (LD) cycle. The second dataset contains 21,414 unannotated transcripts measured at six time points under control (Supplementary Table 2) and desynchronized (Supplementary Table 3) conditions. We BLASTed these transcripts against the known mouse lncRNAs from the NONCODE (http://www.noncode.org/download.php) database and identified 5964 testicular lncRNAs in each dataset (Supplementary Tables 4, 5, and 6). MetaCycle [39] analysis (Supplementary Tables 4, 5, and 6) revealed thousands of rhythmically expressed lncRNAs. We compared the gain and loss of rhythmicity among the lncRNAs under control and desynchronized conditions and analyzed their expression profiles. GO and KEGG analyses revealed the possible involvement of these rhythmically expressed testicular lncRNAs in numerous life processes. Further, we computationally predicted potential orthologs of these rhythmically expressed mouse testicular lncRNAs in humans, rats, and zebrafish, as well as the micropeptides encoded by these conserved lncRNAs, their 3D structures, and functions of the conserved peptides. Our dataset and computational analyses will facilitate functional and experimental investigations of rhythmically expressed testicular lncRNAs in the future.

## 2 Materials and methods

### 2.1 Mouse housing and husbandry

Mice (C57BL/6 J) were raised in a 12:12-hour LD cycle at the Soochow University animal facility. Animal experiments were carried out at the SPF Animal Facility of Soochow University, affiliated with the Association for Assessment and Accreditation of Laboratory Animal Care International. All animal protocols are approved by the Animal Care and Use Committee of the Soochow University (SUDA20230811A01).

### 2.2 Deep sequencing-based transcriptome analyses

We generated two time-course FPKM (Fragments Per Kilobase of transcript per Million mapped reads) datasets of unannotated mouse testicular transcripts. For the 12-time-point dataset, transcriptome analyses of adult mouse testis were conducted at 12 time points, each with two replicates, with a 4-hour interval for a consecutive 48 h under LD conditions. For the six-time-point dataset, transcriptome analyses of adult mouse testis were performed at six time points, each with three replicates, with a four-hour interval for a consecutive 24 h, under desynchronized and control conditions, respectively. We used a paradigm [40] to set a desynchronization model to examine if the peripheral clock in the testis would be altered by shifting light-dark cycles. The mice were synchronized to standard light/dark conditions at 12:12-hour, with lights on at 6 AM and lights off at 6 PM. Subsequently, the mice were assigned to either remain in this lighting regimen or undergo experimental desynchronization. For desynchronization, we subjected mice to serial 12-hour advances of the light/dark cycle every six days for four weeks. Every day of the light shift was preceded and followed by a 12-hour light, 12-hour dark day. RNA-seq-based transcriptome analysis was conducted as previously described [13]. Total RNAs from each sample were extracted with TRIzol (Invitrogen). RNA-sequencing of these testicular transcripts was performed as follows. 1. Sequencing library construction. Sequencing libraries were generated with a total amount of 3 μg RNA per sample and NEBNext®Ultra™ RNA Library Prep Kit (NEB, USA) following the manufacturer’s instructions. 2. Clustering and sequencing. Clustering of the index-coded samples was performed on a cBot Cluster Generation System using TruSeq PE Cluster Kit (Illumia, PE-401–3001) according to the manufacturer’s instructions. After clustering, the library preparations were sequenced on an Illumina Hiseq X 10 platform. 3. Quality control. We calculated the Q20, Q30, GC-content, and duplication data, and then generated the raw reads. All the following analyses were based on clean data of high quality. 4. Transcriptome assembly. Transcriptome assembly was performed as follows: These clean reads were mapped to the mouse genome (GRCm38) with Hisat2 tool software [41]. Only reads with a perfect match or one mismatch were further analyzed and annotated based on the reference genome. Overall, we generated 18,613 unannotated transcripts from our transcriptome analysis of mouse testis for the 12-time-point dataset and 21,414 unannotated transcripts for the six-time-point dataset under control and desynchronized conditions.

### 2.3 Mouse testis RNA-seq datasets

For the 12-time-point dataset, we collected two replicates at 12 time points with a four-hour interval for two consecutive days. Specifically, the data were collected at six time points (ZT0–1, ZT0–2; ZT4–1, ZT4–2; ZT8–1, ZT8–2; ZT12–1, ZT12–1; ZT16–1, ZT16–2; ZT20–1, and ZT20–2) of day 1, and six time points (ZT24–1, ZT24–2; ZT28–1, ZT28–2; ZT32–1, ZT32–2; ZT36–1, ZT36–2; ZT40–1, ZT40–2; ZT44–1, and ZT44–2) of day 2. The 12-time-point data were derived by averaging the expression profiles of the corresponding two replicates as follows: ZT0 = (ZT0–1 + ZT0–2)/2, ZT4 = (ZT4–1 + ZT4–2)/2, ZT8 = (ZT8–1 + ZT8–2)/2, ZT12 = (ZT16–1 + ZT16–2)/2, ZT16 = (ZT20–1 + ZT20–2)/2, and ZT20 = (ZT20–1 + ZT20–2)/2, ZT24 = (ZT24–1 + ZT24-)/2, ZT28 = (ZT28–1 + ZT28–2)/2, ZT32 = (ZT32–1 + ZT32–2)/2, ZT36 = (ZT36–1 + ZT36–2)/2, ZT40 = (ZT40–1 + ZT40–2)/2, and ZT44 = (ZT44–1 + ZT44–2)/2. Together, the 12-time-point dataset included 12 time points with a four-hour interval for 48 consecutive hours, as ZT0, ZT4, ZT8, ZT12, ZT16, ZT20, ZT24, ZT28, ZT32, ZT36, ZT40, and ZT44, each with two replicates (Supplementary Table 1).

For the six-time-point dataset, we measured RNA-seq expression profiles at six time points, each with three replicates, under both control and desynchronized conditions (Supplementary Tables 2 and 3). Specifically, the six time points are ZT0, ZT4, ZT8, ZT12, ZT16, and ZT20. The three replicates for each time point under both control (C) and desynchronized (D) conditions were as follows: for the control (C) condition, ZT0 (ZT0–1-C, ZT0–2-C, and ZT0–3-C); ZT4 (ZT4–1-C, ZT4–2-C, and ZT4–3-C); ZT8 (ZT8–1-C, ZT8–2-C, and ZT8–3-C); ZT12 (ZT12–1-C, ZT12–2-C and ZT12–3-C); ZT16 (ZT16–1-C, ZT16–2-C, and ZT16–3-C); and ZT20 (ZT20–1-C, ZT20–2-C, and ZT20–3-C), and for the desynchronized (D) condition, ZT0 (ZT0–1-D, ZT0–2-D, and ZT0–3-D); ZT4 (ZT4–1-D, ZT4–2-D, and ZT4–3-D); ZT8 (ZT8–1-D, ZT8–2-D, and ZT8–3-D); ZT12 (ZT12–1-D, ZT12–2-D, and ZT12–3-D); ZT16 (ZT16–1-D, ZT16–2-D, and ZT16–3-D); and ZT20 (ZT20–1-D, ZT20–2-D, and ZT20–3-D). The data for each of the six time points were derived by averaging the expression profiles of the corresponding three replicates as follows: for the control (C) condition, ZT0 = (ZT0–1-C + ZT0–2-C + ZT0–3-C)/3, ZT4 = (ZT4–1-C + ZT4–2-C + ZT4–3-C)/3, ZT8 = (ZT8–1-C + ZT8–2-C + ZT8–3-C)/3, ZT12 = (ZT12–1-C + ZT12–2-C + ZT12–3-C)/3, ZT16 = (ZT16–1-C + ZT16–2-C + ZT16–3-C)/3, and ZT20 = (ZT20–1-C + ZT20–2-C + ZT20–3-C)/3; and for the desynchronized (D), ZT0 = (ZT0–1-D + ZT0–2-D + ZT0–3-D)/3, ZT4 = (ZT4–1-D + ZT4–2-D + ZT4–3-D)/3, ZT8 = (ZT8–1-D + ZT8–2-D + ZT8–3-D)/3, ZT12 = (ZT12–1-D + ZT12–2-D + ZT12–3-D)/3, ZT16 = (ZT16–1-D + ZT16–2-D + ZT16–3-D)/3, and ZT20 = (ZT20–1-D + ZT20–2-D + ZT20–3-D)/3. Overall, the six-time-point datasets included six-time points with a four-hour interval for 24 consecutive hours, as ZT0, ZT4, ZT8, ZT12, ZT16, and ZT20, each with three replicates, under control (C) and desynchronized (D) conditions, respectively. These expression profiles have been uploaded on NCBI and can be retrieved with ID PRJNA789131.

### 2.4 Identification of mouse testicular lncRNAs

18,613 unannotated mouse testicular transcripts from the 12-time-point dataset and 21,414 unannotated mouse testicular transcripts from the six-time-point dataset were computationally investigated to identify the potential lncRNAs. We performed BLAST of these transcripts against the known lncRNAs from the NONCODE database with Blast2GO [42] and selected the transcripts that matched with mouse lncRNAs with lower E-value threshold (≤ E-50). In the 12-time-point dataset, out of 18,613 transcripts, 7934 transcripts matched with 5964 NONCODE lncRNAs, indicating that 1970 lncRNAs have more than one transcript profile. In the six-time-point dataset, out of 21,414 transcripts, 8326 transcripts matched with the same 5964 NONCODE lncRNAs, indicating that 2362 lncRNAs have more than one transcript profile. The expression profiles for the lncRNAs with more than one transcript were derived by averaging time-point-specific data of all the shared transcripts for the corresponding lncRNAs. Taken together, we have 5964 lncRNAs with expression profiles in three datasets (Supplementary Tables 4, 5, and 6). Further, since NONCODE lncRNAs’ names lack clarity, we named these 5964 lncRNAs from SUDAMMLNC1 to SUDAMMLNC5964, respectively. The 12-time-point dataset contains 5964 lncRNAs with expression at 12 time points (Supplementary Table 4), while the six-time-point dataset contains the same 5964 lncRNAs with expression at six time points under desynchronized and control conditions (Supplementary Tables 5 and 6). These lncRNAs, along with their expression profiles, enabled us to further investigate their rhythmic expression patterns under different conditions.

### 2.5 Collection of zebrafish testis lncRNAs

We compared the rhythmically expressed lncRNAs in mouse testis with 165 lncRNAs rhythmically expressed in zebrafish testis from our previous study [9].

### 2.6 Identification of rhythmically expressed mouse testicular lncRNAs

We investigated the rhythmicity of 5964 testicular lncRNAs (Supplementary Tables 4, 5, and 6) from three datasets with MetaCycle [39]. LncRNAs with a *P*-value of 0.05 or smaller were considered rhythmic. Out of the 5964 lncRNAs in the 12-time-point dataset, 519 lncRNAs were predicted to be rhythmically expressed (Supplementary Tables 4, 7). In the six-time-point dataset, 475 and 494 lncRNAs were rhythmically expressed under control and desynchronized conditions, respectively (Supplementary Tables 5, 6, 8, and 9). The expression patterns of the rhythmically expressed lncRNAs were visualized using the BioDare2 system (https://biodare2.ed.ac.uk/) [43].

### 2.7 Investigating the possible regulation of rhythmically expressed mouse testicular lncRNAs by E-Box, D-box, or RORE regulatory motifs

*Cis* regulatory motifs have been involved in regulating the rhythmic expression of genes [9], [44], [45]. E-Box, D-Box, and RORE motif elements have been shown to regulate the rhythmic expression of genes peaking in the morning, evening, and night, respectively [46], [47], [48]. Therefore, we classified these rhythmically expressed lncRNAs into morning lncRNAs, evening lncRNAs, and night lncRNAs (Supplementary Tables 7–9) and investigate their potential regulation by *cis* regulatory E-Box, D-Box, or RORE motifs.

In order to find the promoter sequences of these rhythmically expressed lncRNAs, we BLASTed them against the NCBI database and found their corresponding NCBI IDs. Subsequently, we mapped the lncRNA IDs from NCBI (Supplementary Tables 10–12) to the Ensemble IDs using bioDBnet (biological DataBase network) [49]. However, owing to the lack of promoter sequences, we could only find the promoter sequences for a limited number of rhythmically expressed lncRNAs from each dataset. We downloaded the 5000-nucleotide 5′ upstream promoter sequence from Ensembl BioMart. Subsequently, E-Box variable motif CANNTG, D-Box variable motif TTAYGTAA, and RORE variable motif (A/T)A(A/T)NT(A/G)GGTCA (where N can be any nucleotide) in the promoter sequences were computationally analyzed by Find Individual Motif Occurrences (FIMO) [50] with 0.01 or smaller as a fixed *P*-value statistical significance threshold. The probability distribution as input to FIMO for each of the E-Box, D-Box, and RORE motifs was downloaded from JASPAR [51].

### 2.8 GO and KEGG enrichment and annotation of rhythmically expressed mouse testicular lncRNAs

These rhythmically expressed mouse testicular lncRNAs were analyzed with Gene Ontology (GO) [52] and Kyoto Encyclopedia of Genes and Genomes (KEGG) pathway functions. Cytoscape (https://cytoscape.org) [53], an open source Java application, and Ensemble (https://ensembl.org), were used to determine the GO and KEGG pathway annotations.

### 2.9 Principal Component Analysis (PCA) of rhythmically expressed mouse testicular lncRNAs

Principal Component Analysis (PCA) [54] was applied to the time-course expression profiles of these rhythmically expressed lncRNAs to find the two most important principal components representing the corresponding datasets. For each dataset, PCA was also applied to morning lncRNAs, evening lncRNAs, and night lncRNAs, respectively, and assigned a unique score to each of the rhythmically expressed lncRNAs. We selected the lncRNAs with the highest absolute PCA score as the representative lncRNAs to visualize them.

### 2.10 Predicting orthologs of mouse testicular lncRNAs with NCBI BLAST

In order to predict the orthologs of rhythmically expressed mouse testicular lncRNAs in humans, rats, and zebrafish, we downloaded lncRNAs of the human, rat, zebrafish, and human from NONCODE databases [55]. The human, rat, and zebrafish orthologs of rhythmically expressed mouse testicular lncRNAs were determined by the local BLAST program of Blast2GO [42]. Sequences matching with a statistical threshold of E-value ≤ 10 − 50 were considered as potential orthologs.

### 2.11 Predicting secondary structures of the representative mouse testicular lncRNAs

The secondary structures of the representative lncRNAs were computationally predicted using RNAfold web server [56].

### 2.12 Prediction of lncRNA-encoded peptides

CPAT (Coding Potential Alignment Tool) [57] was used to predict the micropetides encoded by conserved mouse testicular lncRNAs, as well as by the zebrafish, rat, and human lncRNAs conserved with these conserved mouse testicular lncRNAs, which are listed in Supplementary Tables 49 to 63.

### 2.13 Predicting 3D structures and functions of lncRNA-encoded peptides

The translation of conserved mouse testicular lncRNAs into amino acid sequences was performed using ExPASy online utility [58]. The 3D structures of the peptides were predicted with (PS)2-v2: Protein Structure Prediction Server [59], [60]. The 3D structures were visualized using Jmol (http://www.jmol.org/).

### 2.14 Analyses of loss-of-rhythmicity, gain-of-rhythmicity, and rhythmicity-maintaining lncRNAs with LimoRhyde

LimoRhyde [61] was used to analyze and visualize the phase and amplitude for the loss- of-rhythmicity, gain-of-rhythmicity, and rhythmicity-maintaining lncRNAs between control and desynchronized conditions.

### 2.15 Calculation of rhythmically expressed testicular lncRNAs uncovered by this study

The study identified 46 lncRNAs rhythmically expressed in both the 12-time-point wild-type dataset and the six-time-point control dataset, 48 lncRNAs rhythmically expressed in both the six-time-point control dataset and the six-time-point desynchronized dataset, and 54 lncRNAs rhythmically expressed in both the 12-time-point dataset and the six-time-point desynchronized dataset. Moreover, 5 lncRNAs are rhythmically expressed in all three datasets. We applied the standard set theory formula n(A ∪ B ∪ C) to compute the total number of rhythmically expressed lncRNAs. The formula is given as.

n(A ∪ B ∪ C) = n(A) + n(B) + n(C) - n(A ∩ B) - n(B ∩ C) - n(A ∩ C) + n(A ∩ B ∩ C),

where.

n(A) = LncRNA rhythmically expressed in the 12-time-point dataset (519);.

n(B) = LncRNA rhythmically expressed in the six-time-point (control) dataset (475);.

n(C) = LncRNA rhythmically expressed in the six-time-point (desynchronized) dataset (494);.

n(A ∩ B) = LncRNA rhythmically expressed in both A and B (46);.

n(B ∩ C) = LncRNA rhythmically expressed in both B and C (48);.

n(A ∩ C) = LncRNA rhythmically expressed in both A and C (54); and.

n(A ∩ B ∩ C) = LncRNA rhythmically expressed in all A, B and C (5).

Hence, the total number of rhythmically expressed lncRNAs = (519 + 475 + 494 – 46 – 48 – 54 + 5) = 1345.

## 3 Results

### 3.1 Rhythmically expressed mouse testicular lncRNAs in the 12-time-point dataset and their GO and KEGG analyses

**Fig. 1:**
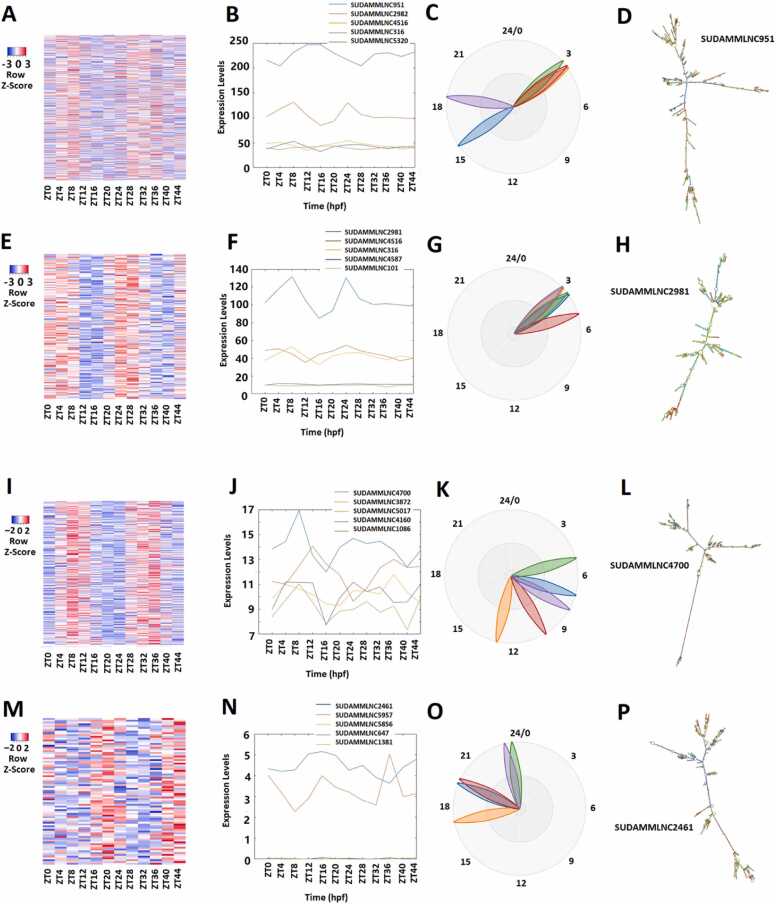
Analyses of 519 rhythmically expressed mouse testicular lncRNAs in the 12-time-point dataset measured over two consecutive days under light/dark (LD) conditions. (A-D) Analysis of all the 519 rhythmically expressed mouse testicular lncRNAs: Heat map (A) of all the 519 rhythmically expressed lncRNAs, expression profiles (B) and phases (C) of representative lncRNAs, and secondary structure plot of a representative lncRNA (D). (E-H) Analysis of 171 morning lncRNAs: Heat map of the 171 morning lncRNAs (E), expression profiles (F) and phases (G) of representative morning lncRNAs, and secondary structure plot of a representative lncRNA (H). (I-L) Analysis of 266 evening lncRNAs: Heat map of the 266 evening lncRNAs (I), expression profiles (J) and phases (K) of representative evening lncRNAs, and secondary structure plot of a representative evening lncRNA (L). (M-P) Analysis of 82 night lncRNAs: Heat map of the 82 night lncRNAs (M), expression profiles (N) and phases (O) of representative evening lncRNAs, and secondary structure plot of a representative night lncRNA (P).

Our previous study classified rhythmically expressed lncRNAs into morning lncRNAs, evening lncRNAs, and night lncRNAs [9], [31]. The rhythmicity analysis of gene expression profiles of 5964 mouse testicular lncRNAs from the 12-time-point dataset (Fig. 1A-D; Supplementary Tables 4 and 7) with MetaCycle revealed 519 (approximately 8.7%) rhythmically expressed mouse testicular lncRNAs. The heat map shows their expression levels at the 12 time points (Fig. 1A), while the BioDare2 plots (Fig. S3A) show the phases of these rhythmically expressed lncRNAs. The two most important principal components of these 519 lncRNAs were determined by PCA (Fig. S2A). The representative lncRNAs, their BioDare2 plots, and secondary structures are also shown (Fig. 1B-1D and Fig. S3B-S3E).

Subsequently, based on their peak expression patterns, these 519 rhythmically expressed mouse testicular lncRNAs were classified into 171 lncRNAs peaked in the morning (ZT0/ZT24 and ZT4/ZT28), 266 lncRNAs peaked in the evening (ZT8/ZT32 and ZT12/ZT36), and 82 lncRNAs peaked in the night (ZT16/ZT40 and ZT20/ZT44) (Supplementary Table 7). PCA was performed for these morning lncRNAs (Fig. S2B), evening lncRNAs (Fig. S2C), and night lncRNAs (Fig. S2D). Because the first principal component captures most of the variance in the datasets, these lncRNAs were ranked based on the corresponding absolute PCA scores from the first principal component. The heat map with expression levels at 12 time points, PCA with two most important principal components, representative lncRNAs with expression levels, BioDare2 plots with phases, and secondary structures are shown for the morning lncRNAs (Fig. 1E-1 H, Fig. S2B, and Fig. S3F-S3J), evening lncRNAs (Fig. 1I-1 L, Fig. S2C, and Fig. S3K-S3O), and night lncRNAs (Fig. 1M-1 P, Fig. S2D, and Fig. S3P-S3T), respectively. These morning lncRNAs, evening lncRNAs, and night lncRNAs exhibit distinct phases, expression patterns, and secondary structures, as shown by the heat maps (Figs. 1E, 1I, and 1M) and BioDare2 plots (Fig. S3F, S3K, and S3P).

E-box, D-box, and RORE elements have been shown to mediate the expression of rhythmically expressed lncRNAs [9], [44], [45]. We interrogated E-box, D-box, or RORE motifs of rhythmically expressed 171 morning, 276 evening, and 82 night mouse testicular lncRNAs, respectively. However, due to the lack of the promoter sequences of mouse testicular lncRNAs, we could only find promoter sequences of 16 morning lncRNAs, 20 evening lncRNAs, and five night lncRNAs (Supplementary Table 10). We searched these promoter sequences for E-box variable motif CANNTG, D-box variable motif TTAYGTAA, or RORE promoter motif (A/T)A(A/T)NT(A/G)GGTCA with FIMO [50]. Interestingly, each of the 16 morning lncRNAs, 20 evening lncRNAs, and five night lncRNAs contains E-box, D-box, or RORE promoter motifs, respectively (Supplementary Tables 13–15).

**Fig. 2:**
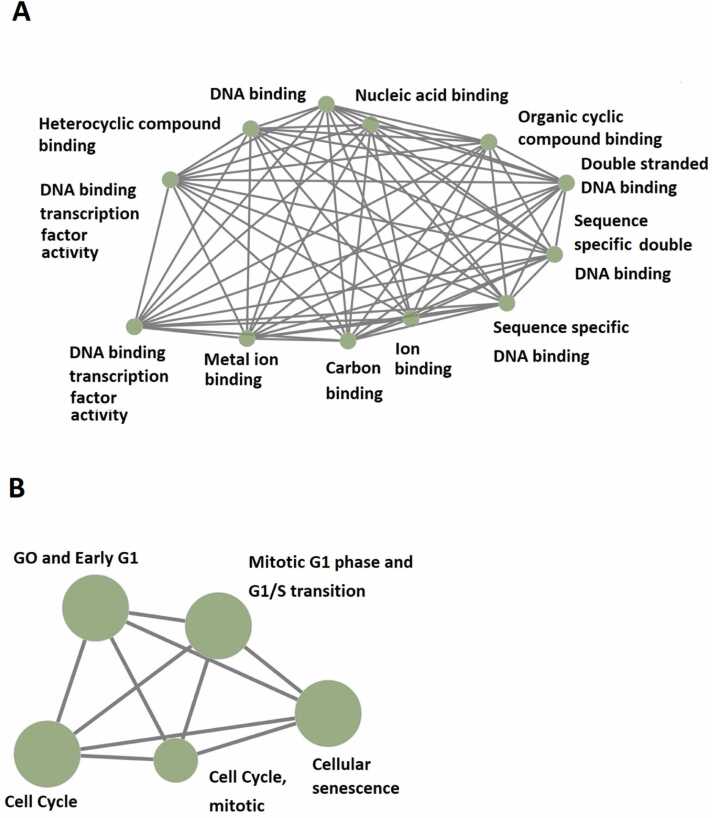
GO and KEGG analyses of 519 rhythmically expressed mouse testicular lncRNAs in the 12-time-point dataset measured over two consecutive days under light/dark (LD) conditions. (A) GO analysis revealed the potential involvement of some of these rhythmically expressed mouse testicular lncRNAs in numerous biological processes, such as DNA binding, Nucleic acid binding, Organic cyclic compound binding, Double-stranded DNA binding, Heterocyclic compound binding, DNA binding transcription factor activity, and Metal ion binding. (B) KEGG enrichment analysis shows the possible involvement of some of these rhythmically expressed mouse testicular lncRNAs in mitotic GO and Early G1, Mitotic G1 phase and G1/S transition, and Cellular senescence.

Next, GO and KEGG analyses of these rhythmically expressed mouse testicular lncRNAs were conducted (Fig. 2, Supplementary Tables 16–18). GO analyses revealed that as many as eight morning lncRNAs (Supplementary Tables 16), ten evening lncRNAs (Supplementary Table 17), and four night lncRNAs (Supplementary Table 18) are possibly involved in numerous biological functions, including gene expression (GO:0010467) and molecular function (GO:0003674). KEGG analyses revealed that these lncRNAs are likely involved in several fundamental biological processes, including regulation of cell cycle and G1/S transition (Fig. 2).

Taken together, we identified hundreds of mouse testicular rhythmically expressed lncRNAs with certain possible GO and KEGG functions, which are likely regulated by the E-box, D-box, or RORE in their promoter regions. Although additional experimental verification is required to confirm these bioinformatic findings, our computational predictions support the previous hypothesis [9], [44], [45] of regulation of rhythmically expressed lncRNAs by the promoter motifs, and their involvement in various biological functions.

### 3.2 Rhythmically expressed mouse testicular lncRNAs in the six-time-point control dataset and their GO and KEGG analyses

**Fig. 3:**
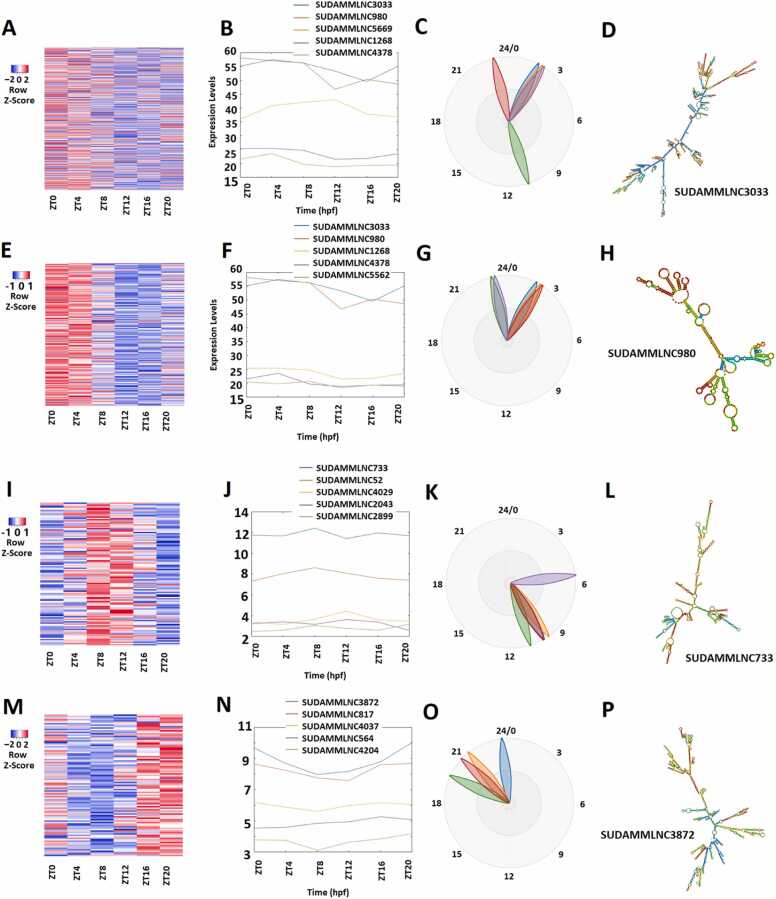
Analyses of 475 rhythmically expressed mouse testicular lncRNAs in the six-time-point control dataset measured over one day under light-dark (LD) conditions. (A-D) Analysis of all the 475 rhythmically expressed mouse testicular lncRNAs: Heat map (A) of all the 475 rhythmically expressed lncRNAs, expression profiles of representative lncRNAs (B) and phases of representative lncRNAs (C), and secondary structure plot (D) of a representative lncRNA. (E-H) Analysis of 264 morning lncRNAs: Heat map of the 264 morning lncRNAs (E), expression profiles of representative morning lncRNAs (F) and phases of representative morning lncRNAs (G), and secondary structure plot of a representative lncRNA (H). (I-L) Heat map of the 112 evening lncRNAs (I), expression profiles of representative evening lncRNAs (J) and phases of representative evening lncRNAs (K), and secondary structure plot of a representative evening lncRNA (L). (M-P) Heat map of the 99 night lncRNAs (M), expression profiles of representative evening lncRNAs (N) and phases of representative night lncRNAs (O), and secondary structure plot of a representative night lncRNA (P).

We also performed the rhythmicity analysis of gene expression profiles of 5964 mouse testicular lncRNAs from the six-time-point control dataset (Fig. 3A-3D; Supplementary Tables 5 and 8) with MetaCycle and identified 475 (approximately 8%) rhythmically expressed mouse testicular lncRNAs. The heat map shows the expression profiles of these 475 lncRNAs at the six time points (Fig. 3A), and the BioDare2 plots (Fig. S5A) depict the distinct phases for these rhythmically expressed lncRNAs. Further, PCA shows the two most important principal components of these 475 lncRNAs (Fig. S4A). The representative lncRNAs, their BioDare2 plots, and secondary structures are also shown (Fig. 3B-3D and Fig. S5B-S5E).

These 475 lncRNAs were classified into 264 lncRNAs peaked in the morning (ZT0 and ZT4), 112 lncRNAs peaked in the evening (ZT8 and ZT12), and 99 lncRNAs peaked in the night (ZT16 and ZT20) (Supplementary Table 14), respectively. PCA of these morning lncRNAs (Fig. S4B), evening lncRNAs (Fig. S4C), and night lncRNAs (Fig. S4D) allowed for ranking them based on the corresponding absolute PCA scores from the first principal components. The expression levels in the form of the heat map, the first two most important principal components from PCA, representative lncRNAs, phases predicted by BioDare2, and secondary structures are shown for the morning lncRNAs (Fig. 3E-3 H, Fig. S4B, and Fig. S5F-S5J), evening lncRNAs (Fig. 3I-3 L, Fig. S4C, and Fig. S5K-S5O), and night lncRNAs (Fig. 3M-3 P, Fig. S4D, and Fig. S5P-S5T), respectively. As shown by the expression levels in the heat maps (Figs. 3E, 3I, and 3M) and the phases depicted by the BioDare2 plots (Fig. S5F, S5K, and S5P), these morning lncRNAs, evening lncRNAs, and night lncRNAs exhibit distinct expression patterns, phases, and secondary structures.

Next, we interrogated E-box, D-box, and RORE motifs of these rhythmically expressed 264, 112, and 99 mouse testicular lncRNAs that peaked in the morning, the evening, and the night, respectively. We could only find promoter sequences of 28 morning lncRNAs, six evening lncRNAs, and 13 night lncRNAs (Supplementary Table 11), and observed that each of the 28 morning lncRNAs, six evening lncRNAs, and 13 night lncRNAs contains E-box, D-box, or RORE motifs, respectively (Supplementary Tables 19–21).

GO analysis (Fig. S6A) revealed that as many as 13 morning lncRNAs (Supplementary Tables 22), three evening lncRNAs (Supplementary Table 23), and seven night lncRNAs (Supplementary Table 24) are likely involved in numerous biological functions, including miRNA-mediated gene silencing (GO:0035195), cellular_component (GO:0005575), and RNA processing (GO:0006396). KEGG analysis revealed that the lncRNAs are possibly involved in several fundamental biological processes, including regulation of glutamatergic synapse, cytoplasm, and postsynaptic actin cytoskeleton (Fig. S6B).

### 3.3 Analyses of 46 rhythmically expressed lncRNAs shared between the 12-time-point and six-time-point datasets

Intriguingly, although the 12-time-point dataset and six-time-point control dataset shared the same 5964 lncRNAs, the number of rhythmically expressed lncRNAs in each dataset differs significantly. In particular, the 12-point-time dataset has 44 more rhythmically expressed lncRNAs than the six-time-point control dataset, which could be due to the differing sampling strategies [62], [63]. However, 46 lncRNAs are rhythmically expressed under both the 12-time-point and six-time-point conditions (Supplementary Tables 25 and 26). Hence, we further divided these 46 lncRNAs into morning, evening, and night groups based on the expression patterns from the 12-time-point or six-time-point conditions and visualized the expression patterns of these lncRNAs, respectively.

**Fig. 4:**
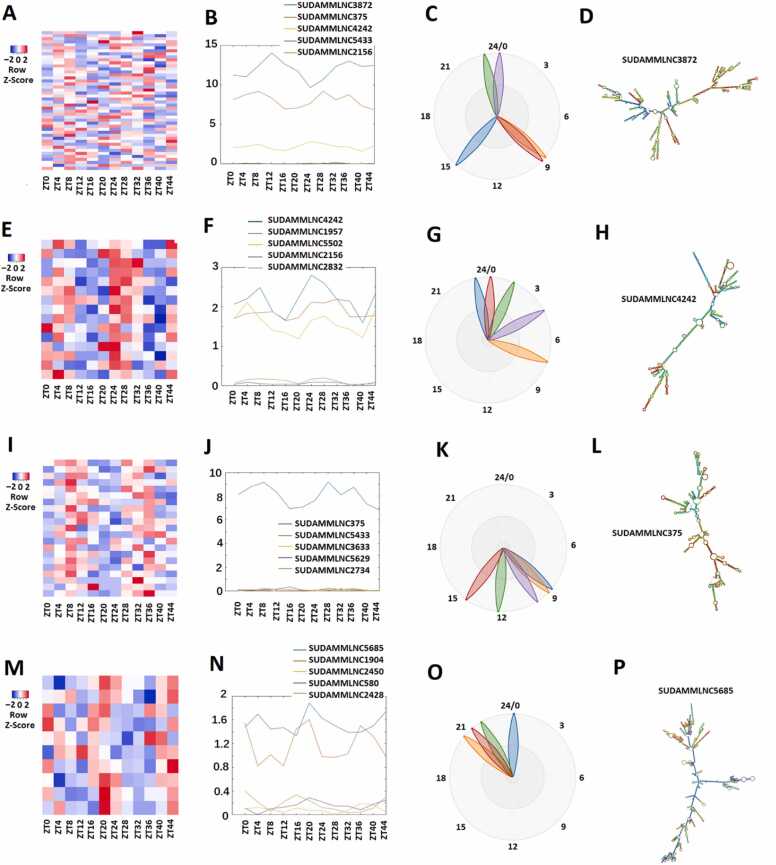
Analysis of 46 rhythmically expressed mouse testicular lncRNAs shared between the 12-point-time and 6-point-time control datasets using the 12-time-point data. (A-D) Analysis of all the 46 rhythmically expressed mouse testicular lncRNAs from the 12-time-point dataset: Heat map (A), expression profiles (B), and phases (C) of all the 46 rhythmically expressed lncRNAs, and secondary structure plot of a representative lncRNA (D). (E-H) Analysis of 15 rhythmically expressed morning lncRNA: Heat map (E), expression profiles (F), and phases (G) of the 15 rhythmically expressed morning lncRNAs, and secondary structure plot of a representative lncRNA (H). (I-L) Analysis of 21 rhythmically expressed evening lncRNAs: Heat map (I), expression profiles (J), and phases (K) of the 21 rhythmically expressed evening lncRNAs, and secondary structure plot of a representative lncRNA (L). (M-P) Analysis of 10 rhythmically expressed night lncRNAs: Heat map (M), expression profiles (N), and phases (O) of the 10 rhythmically expressed night lncRNAs, and secondary structure plot of a representative evening lncRNA (P).

The heat map, Biodare2 plots, PCA analyses, and representative lncRNAs of the 46 lncRNAs from the 12-time-point condition are shown (Fig. 4, Fig. S7-S8, and Supplementary Table 25). The analyses of these 46 lncRNAs (Fig. 4A-4D, Fig. S7A, Fig. S8A-S8E) from the 12-time-point condition revealed 15 lncRNAs, 21 lncRNAs, and ten lncRNAs with peak expression in the morning (Fig. 4E-4 H, Fig. S7B, Fig. S8F-S8J), the evening (Fig. 4I-4 L, Fig. S7C, Fig. S8K-S8O), and the night (Fig. 4M-4 P, Fig. S7D, Fig. S8P-S8T), respectively.

A similar analysis of these 46 lncRNAs (Fig. S9-S11) using the expression profiles from the six-time-point control dataset revealed 25 morning lncRNAs (Fig. S9E-S9H, Fig. S10B, Fig. S11F-S12J), 11 evening lncRNAs (Fig. S9I-S9L, Fig. S10C, Fig. S11K-S12O), and 10 night lncRNAs (Fig. S9M-S9P, Fig. S10D, Fig. S11P- S11T).

### 3.4 Rhythmically expressed mouse testicular lncRNAs in the six-time-point desynchronized dataset and their GO and KEGG analyses

Rhythmicity analysis of gene expression profiles of 5964 mouse testicular lncRNAs from the six-time-point desynchronized dataset (Fig. S12A-S12D; Supplementary Table 6) identified 494 (approximately 8.29%) rhythmically expressed mouse testicular lncRNAs. The heat map shows the expression profiles of these 494 lncRNAs at six time points (Fig. S12A), while the BioDare2 plot (Fig. S13A) shows the phases of these rhythmically expressed lncRNAs. PCA analyses (Fig. S14A) show the two most important principal components of these 494 lncRNAs. The phases of representative lncRNAs computationally predicted by the BioDare2 (Fig. S5B-S5D), and their secondary structures are shown (Fig. S13B-S13E).

These 494 lncRNAs (Supplementary Table 9) were classified into 200 morning (ZT0 and ZT4) lncRNAs, 191 evening (ZT8 and ZT12) lncRNAs, and 103 night (ZT16 and ZT20) lncRNAs, which were ranked based on the corresponding absolute PCA scores from the first principal components of their PCA analyses (Fig. S14B-S14D). The heat map, PCA analyses, representative lncRNAs, BioDare2 plots, and secondary structures are shown for the morning lncRNAs (Fig. S12E-S12H, Fig. S13B, and Fig. S14F-S14J), evening lncRNAs (Fig. S12I-S12L, Fig. S13C, and Fig. S14K-S14O), and night lncRNAs (Fig. S12M-S12P, Fig. S13D, and Fig. S14P-S14T) respectively. These morning lncRNAs, evening lncRNAs, and night lncRNAs exhibit distinct expression patterns under the desynchronized condition, as shown by the heat maps (Fig. S12E, S12I, and S12M) and BioDare2 plots (Fig. S13F, S13K, and S13P).

We then analyzed the E-box, D-box, and RORE motifs of these 200, 191, and 103 mouse testicular rhythmically expressed lncRNAs that peaked in the morning, the evening, and the night, respectively. We could only find the regulatory motifs from 24 morning lncRNAs, 14 evening lncRNAs, and seven night lncRNAs (Supplementary Table 12), which contain E-box, D-box, and RORE motifs, respectively (Supplementary Tables 27–29).

GO and KEGG analyses of these rhythmically expressed mouse testicular lncRNAs were also conducted (Fig. S15, Supplementary Tables 30–32). While GO analysis revealed that as many as 15 morning lncRNAs (Supplementary Table 30), 11 evening lncRNAs (Supplementary Table 31), three night lncRNAs (Supplementary Table 32) are likely involved in numerous biological functions, including skeletal system development (GO:0001501), tissue homeostasis (GO:0001894), and gene expression (GO:0010467), KEGG analysis revealed that the lncRNAs are possibly involved in several fundamental biological processes, including protein ubiquitination, metabolic process, and macromolecule modification (Fig. S15).

### 3.5 Analyses of 427 mouse testicular lncRNAs that lost rhythmicity in desynchronized conditions

**Fig. 5:**
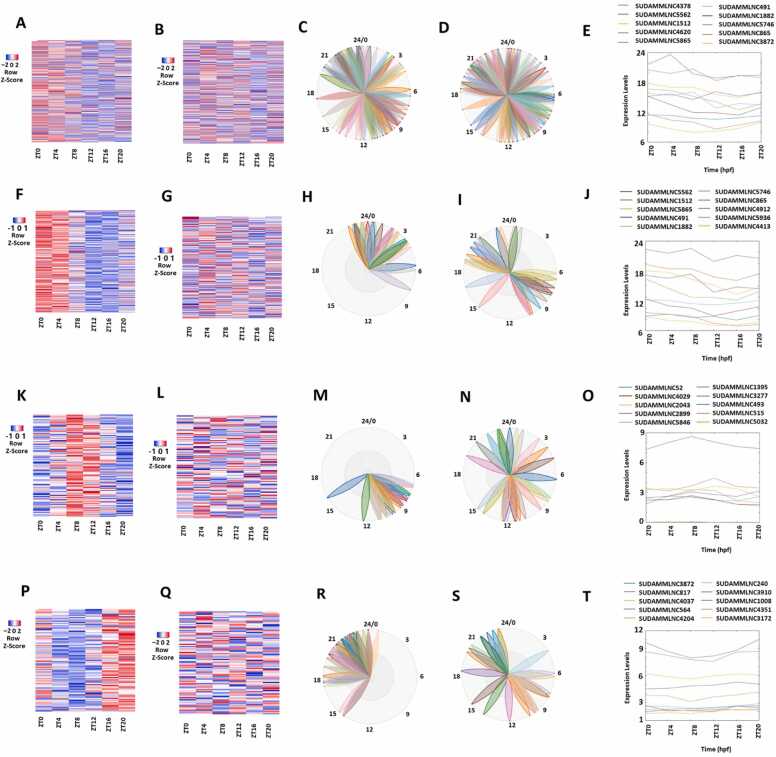
Analyses of the 427 mouse testicular lncRNAs that lost rhythmicity in desynchronized conditions with data from both control and desynchronized conditions. (A, C) Heat map (A) and Biodare2 plot (C) of all the 427 lncRNAs with data from the 6-point-time control dataset. (B, D) Heat map (B) and Biodare2 plot (D) of all the 427 lncRNAs with data from the 6-point-time desynchronized dataset. (E) Expression profiles of top-ten representative lncRNAs from the control condition of the overall 427 lncRNAs. (F, H) Heatmap (F) and Biodare2 plot (H) of the 234 morning lncRNAs with data from the 6-point-time control dataset. (G, I) Heat map (G) and Biodare2 plot (I) of the 234 morning lncRNAs with data from the 6-point-time desynchronized dataset. (J) Expression profiles of top-ten representative lncRNAs from the control condition of the 234 morning lncRNAs. (K, M) Heat map (K) and Biodare2 plot (M) of the 104 evening lncRNAs with data from the 6-point-time control dataset. (L, N) Heatmap (L) and Biodare2 plot (N) of the 104 evening lncRNAs with data from the 6-point-time desynchronized dataset. (O) Expression profiles of top-ten representative lncRNAs from the control condition of the 104 evening lncRNAs. (P, R) Heat map (P) and Biodare2 plot (R) of the 89 night lncRNAs with data from the six-time-point control dataset. (Q, S) Heatmap (Q) and Biodare2 plot (S) of the 89 night lncRNAs with data from the 6-point-time desynchronized dataset. (T) Expression profiles from both control and desynchronized conditions of the top-ranked representative lncRNAs from the 89 night lncRNAs.

**Fig. 6:**
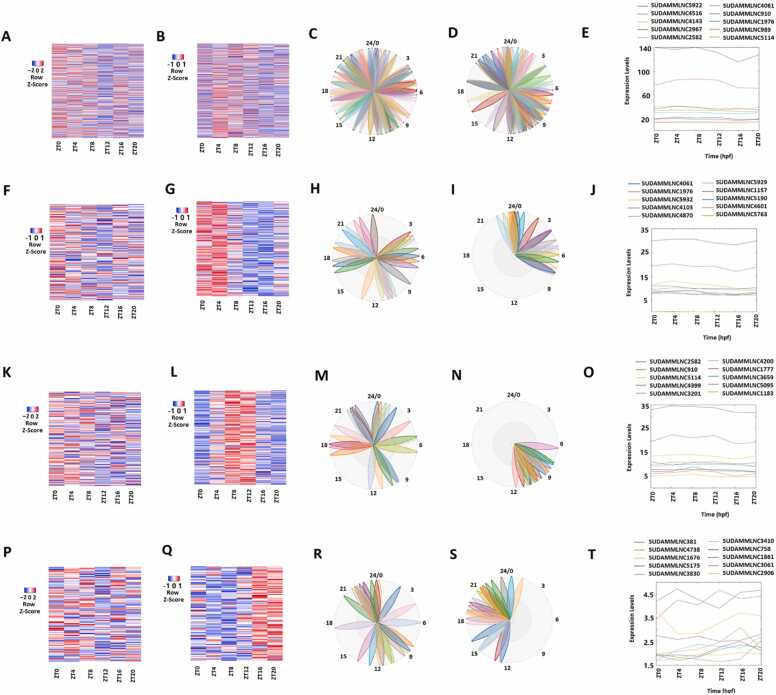
Analyses of the 446 mouse testicular lncRNAs that gained rhythmicity in desynchronized conditions with data from both control and desynchronized conditions. (A, C) Heat map (A) and phases (C) of all the 446 lncRNAs from the 6-point-time control dataset. (B, D) Heat map (B) and phases (D) of all the 427 lncRNAs from the 6-point-time desynchronized dataset. (E) Expression profiles of top-ten representative lncRNAs from the desynchronized condition of the overall 446 lncRNAs. (F, H) Heat map (F) and phases (H) of the 174 morning lncRNAs from the 6-point-time control dataset. Heat map (G) and phases (I) of the 174 morning lncRNAs with data from the 6-point-time desynchronized dataset. (J) Expression profiles of top-ten representative lncRNAs from the desynchronized condition of the 174 morning lncRNAs. (K, M) Heat map (K) and phases (M) of the 196 evening lncRNAs with data from the 6-point-time control dataset. (L, N) Heat map (L) and phases (N) of the 196 evening lncRNAs with data from the 6-point-time desynchronized dataset. (O) Expression profiles of top-ten representative lncRNAs from the desynchronized condition of the 196 evening lncRNAs. (P, R) Heat map (P) and phases (R) of the 96 night lncRNAs with data from the 6-point-time control dataset. (Q, S) Heat map (Q) and phases (S) of the 96 night lncRNAs with data from the 6-point-time desynchronized dataset. (T) Expression profiles of top-ten representative lncRNAs from the desynchronized condition of the 196 night lncRNAs.

**Fig. 7:**
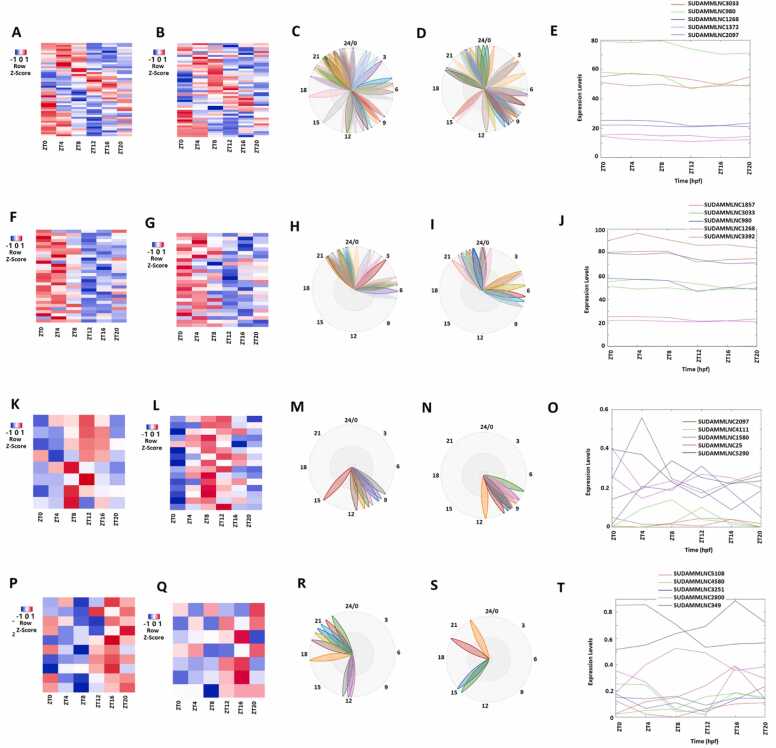
Analyses of the 48 mouse testicular lncRNAs that maintained between the control and desynchronized conditions using the 6-point-time control and desynchronized datasets. (A, C) Heat map (A) and phases (C) of all the 48 lncRNAs from the 6-point-time control dataset. (B, D) Heat map (B) and phases (D) of all the 48 lncRNAs from the 6-point-time desynchronized dataset. (E) Expression profiles the top-five representative lncRNAs, each from both control (C) and desynchronized (D) conditions of the overall 48 lncRNAs. (F, H) Heat map (F) and phases (H) of the morning lncRNAs with data from the 6-point-time control dataset. (G, I) Heat map (G) and phases (I) of the morning lncRNAs with data from the 6-point-time desynchronized dataset. (J) Expression profiles of the top-five representative lncRNAs, each from both control (C) and desynchronized (D) conditions of the morning lncRNAs. (K, M) Heat map (K) and phases (M) of the evening lncRNAs with data from the 6-point-time control dataset. (L, N) Heat map (L) and phases (N) of the evening lncRNAs with data from the 6-point-time desynchronized dataset. (O) Expression profiles of the top-five representative lncRNAs, each from both control (C) and desynchronized (D) of the evening lncRNAs. (P, R) Heat map (P) and phases (R) of the night lncRNAs with data from the 6-point control dataset. (Q, S) Heat map () and phases (S) of the night lncRNAs with data from the 6-point desynchronized dataset. (O) Expression profiles of the top-five representative lncRNAs, each from both control (C) and desynchronized (D) conditions of the night lncRNAs.

**Fig. 8:**
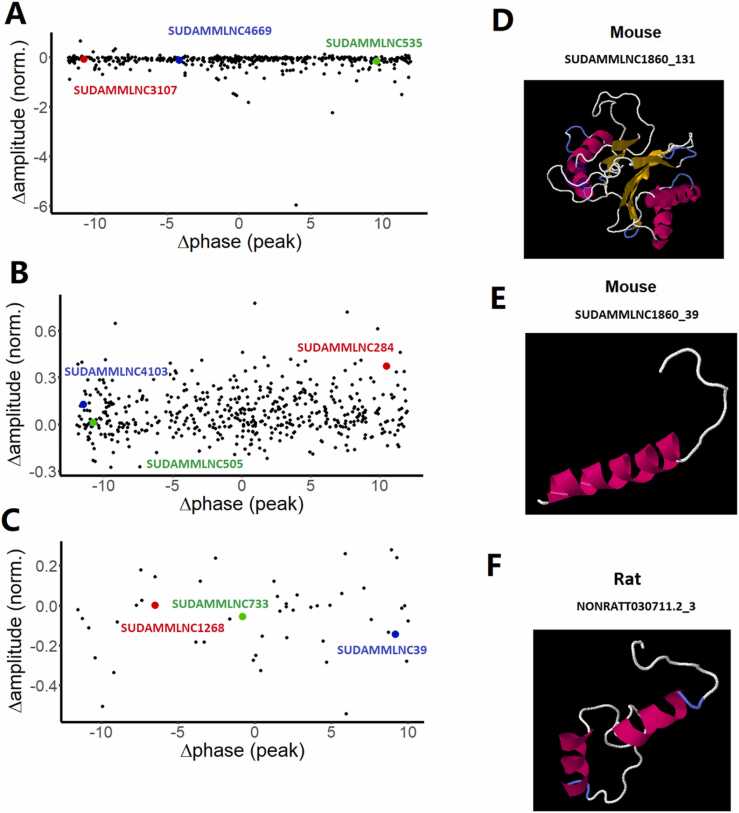
Visualization of changes of the phases and amplitudes of loss-of-rhythmicity, gain-of-rhythmicity, and rhythmicity-maintaining lncRNAs between control and desynchronized conditions, and 3-dimensional structures of the conserved lncRNA-encoded peptides. (A-C) Changes in the phases and amplitudes of loss-of-rhythmicity l mouse ncRNAs (A) and gain-of-rhythmicity mouse lncRNAs in the desynchronized condition (B), and rhythmicity-maintaining mouse lncRNAs between control and desynchronized conditions (C). (D-F) 3D models of peptides encoded by two mouse testicular lncRNAs (SUDAMMLNC1860_131, and SUDAMMLNC1860_39) (D, E) conserved with one rat lncRNA (NONRATT030711.2_3) (F). The predicted 3D models of the conserved lncRNA-encoded peptides revealed the presence of α-helix (pink or purple motif structure), β-strand (yellow layered band), and random coils (white or blue thread) with the known domains from Protein Data Bank, such as D (1g3kA), E (2axtL), and F (1g5cA).

Our previous studies have shown that the lncRNAs may alter their rhythmic expression patterns under different treatment conditions [9], [31]. We investigated the loss and gain of rhythmicity mouse testicular lncRNAs between the control and desynchronized conditions (Supplementary Tables 33–37, Fig. 5, Fig. 6, Fig. 7, Fig. 8, and Fig. S16-S23). Interestingly, we found that 427 rhythmically expressed lncRNAs in the control condition became arrhythmic in the desynchronized condition (Figs. 5, 8A, Fig. S16-S17), suggesting a potential loss of rhythmicity (Fig. 8A). For example, the lncRNA SUDAMMLNC4378 has *P*-values of 0.003395634 27 in the control condition but 0.9441829 in the desynchronized condition (Supplementary Table 38).

These 427 rhythmically expressed lncRNAs in the control condition were classified into 234 morning lncRNAs (Figs. 5F, 5H), 104 evening lncRNAs (Fig. 5K-5 M), and 89 night lncRNAs (Fig. 5P-5R). These 427 lncRNAs (Fig. S16A), as well as 234 morning lncRNAs (Fig. S16B), 104 evening lncRNAs (Fig. S16C), and 89 night lncRNAs (Fig. S16D), were ranked based on the corresponding absolute PCA scores from the first principal components of their PCA analyses. The heat map, PCA analyses, and Biodare2 plots of the expression levels of these 427 lncRNAs are shown (Figs. 5A, 5C, Fig. S16A), respectively. The representative lncRNAs and secondary structures are visualized (Fig. 5E and Fig. S17A-SE). Similar analyses are conducted for the 234 morning lncRNAs, the 104 evening lncRNAs (Figs. 5K, 5M, 5O, Fig. S16C, and Fig. S17K-S17O), and the 89 night lncRNAs (Figs. 5P, 5R, 5T, Fig. S16D, and Fig. S17P-S17T), respectively. The heat maps (Figs. 5F, 5K, and 5P) and BioDare2 plots (Figs. 5H, 5M, and 5R) of these morning lncRNAs, evening lncRNAs, and night lncRNAs exhibit different expression patterns and phases.

We jointly visualized the 427 lncRNAs (Fig. 5) with both rhythmically expressed data from the control condition and non-rhythmically expressed data from the desynchronized condition. The plots with non-rhythmically expressed desynchronized data are shown in the heat maps of all 427 lncRNAs (Fig. 5B), morning lncRNA (Fig. 5G), evening lncRNAs (Fig. 5L), and night lncRNAs (Fig. 5Q). Phases of all 427 lncRNAs (Fig. 5D), morning lncRNA (Fig. 5I), evening lncRNAs (Fig. 5N), and night lncRNAs (Fig. 5S), and their representative lncRNAs are shown for all 427 lncRNAs (Fig. 5E), morning lncRNA (Fig. 5J), evening lncRNAs (Fig. 5O) and night lncRNAs (Fig. 5T). The combined plots of 427 lncRNAs (Fig. 5) with both rhythmically expressed data from the control condition and non-rhythmically expressed data from the desynchronized condition clearly demonstrated the loss of rhythmicity from the control to desynchronized conditions.

### 3.6 Analyses of 446 mouse testicular lncRNAs that gained rhythmicity in desynchronized conditions

In contrast, 446 arrhythmic lncRNAs (Figs. 6, 8B, Fig. S18-S19) in the control condition turned up rhythmic in the desynchronized condition, suggesting a possible gain of rhythmicity. For instance, the lncRNA SUDAMMLNC5922 has *P*-values of 0.713860868 in the control condition but 0.011600656 in the desynchronized condition (Supplementary Table 38).

These 446 rhythmically expressed lncRNAs in the desynchronized condition were grouped into 174 morning lncRNAs (Figs. 6G, 6I, 6J, Fig. S18B, Fig. S19F-19 J), 176 evening lncRNAs (Figs. 6L, 6N, 6O, Fig. S18C, Fig. S19F-19 J), and 96 night lncRNAs (Figs. 6Q, 6S, 6T, Fig. S18D, Fig. S19K-19 O). The PCA analyses of all 427 lncRNAs (Fig. S18A), 174 morning lncRNAs (Fig. S18B), 176 evening lncRNAs (Fig. S18C), and 96 night lncRNAs (Fig. S18D) allowed for ranking them based on the corresponding absolute PCA scores from the first principal components. We analyzed the expression levels of the all 446 lncRNAs, 174 morning lncRNAs, 176 evening lncRNAs, and 96 night lncRNAs (Fig. S18-S20). The analysis of all 446 lncRNAs in the form of expression levels, data variance captured by the first two principal components from the PCA, and BioDare2 phase plots has been illustrated (Figs. 6B, 6D, and Fig. S18A, Fig. S19A-19E), respectively. The representative lncRNAs are analyzed in the form of expression levels (Fig. 6E), BioDare2 phase plots (Fig. 6D), and secondary structures (Fig. S19A-S19D).

We jointly visualized the 446 lncRNAs (Fig. 6) with both non-rhythmically expressed data from the control condition and rhythmically expressed data from the desynchronized condition. The plots of the non-rhythmically expressed control data are shown in the heat map of all arrhythmically expressed 446 lncRNAs (Fig. 6A), morning lncRNA (Fig. 6F), evening lncRNAs (Fig. 6K) and night lncRNAs (Fig. 6P), phases of all 446 lncRNAs (Fig. 6C), morning lncRNAs (Fig. 6H), evening lncRNAs (Fig. 6M), and night lncRNAs (Fig. 6R), and their representative lncRNAs are shown for all 446 lncRNAs (Fig. 6E), morning lncRNA (Fig. 6J), evening lncRNAs (Fig. 6O), and night lncRNAs (Fig. 6T). The combined plots of these 446 lncRNAs (Fig. 6) with both non-rhythmically expressed data from the control condition and rhythmically expressed data from the desynchronized condition clearly demonstrated the gain of rhythmicity from the control to desynchronized conditions.

### 3.7 Analyses of 48 rhythmicity-maintaining mouse testicular lncRNAs between control and desynchronized conditions

Intriguingly, 48 lncRNAs maintained rhythmicity under both the control and desynchronized conditions (Figs. 7, 8C, Fig. S20-23). For example, the lncRNA SUDAMMLNC3033 has *P*-values of 0.041769182 and 0.001412983 in the control and desynchronized conditions (Supplementary Table 38), respectively.

These 48 rhythmicity-maintaining mouse lncRNAs were analyzed using data from both control and desynchronized conditions (Fig. 7A-7D, Supplementary Tables 36–38), and the corresponding representative lncRNAs were analyzed with the PCA analyses (Fig. S20, S22). The analyses of the 48 rhythmically expressed lncRNAs (Fig. 7A) using the six-time-point data from the control and desynchronized conditions revealed that although these 48 lncRNAs maintained rhythmicity under both control and desynchronized conditions, their phases, periods, and amplitudes varied under the two conditions (Supplementary Table 38). Between control and desynchronized conditions, most of these 48 lncRNAs displayed either advance or delay of phases, either lengthened or shortened periods, and/or either increase or decrease of amplitudes. For example, the lncRNA SUDAMMLNC3033 has a phase and amplitude of 5.017012835 and 2.853012515 under the control conditions, whereas under the desynchronized condition, its phase and amplitude change into 8.093165742 and 2.400033621, respectively.

We classified these 48 lncRNAs into the morning lncRNAs, the evening lncRNAs, and the night lncRNAs with expression profiles from both control and desynchronized conditions.

Using the control data, the 48 lncRNAs were categorized into 30 morning lncRNAs, eight evening lncRNAs, and ten night lncRNAs (Figs. 7F, 7K and 7P). The analysis in the form of heat map, PCA analyses, and secondary structures for the 48 lncRNAs (Figs. 7A, 7C, 7E, Fig. S20A, and Fig. S21A-S21E), the morning lncRNAs (Figs. 7F, 7H, 7O, Fig. S20B, and Fig. S21F-S21J), evening lncRNAs (Figs. 7K, 7M, 7O, Fig. S20C, and Fig. S21K-S21O), and the night lncRNAs (Figs. 7P, 7R, 7T, Fig. S20D, and Fig. S21P-S21T) elucidated distinct expression patterns for these lncRNAs.

The analyses of the 48 rhythmically expressed lncRNAs (Figs. 7B, 7D, 7E, Fig. S22A, and Fig. S23A-S23E) using the six-time-point data from the desynchronized condition revealed that out of the 48 lncRNAs, 26 lncRNAs peaked in the morning (Figs. 7G, 7I, 7J, Fig. S22B, and Fig. S23F-S23J), 15 lncRNAs peaked in the evening (Figs. 7L, 7N, 7O, Fig. S22B, and Fig. S23K-S23O), and seven lncRNAs peaked in the night (Figs. 7Q, 7S, 7T, Fig. S22D, and Fig. S23P-S23T). PCA analyses of all 48 lncRNAs (Fig. S22A), 26 morning lncRNAs (Fig. S22B), 15 evening lncRNAs (Fig. S22C), and seven night lncRNAs (Fig. S22D) allowed for ranking them based on the corresponding absolute PCA scores from the first principal components. Analyses of the lncRNAs’ expression levels in the form of heat maps (Figs. 7G, 7L, 7Q) and BioDare2 plots (Figs. 7I, 7N, 7S) revealed distinct expression patterns of these morning lncRNAs, evening lncRNAs, and night lncRNAs.

Further, we visualized the correlation of phases and amplitude for the loss-of- rhythmicity lncRNAs between control and desynchronized datasets (Fig. 8A), gain-of-rhythmicity lncRNAs between control and desynchronized datasets (Fig. 8B), and rhythmicity-maintaining lncRNAs under both control and desynchronized conditions (Fig. 8C) with LimoRhyde. The phase and amplitude correlation clearly demonstrated differing correlations among the loss-of-rhythmicity, gain-of-rhythmicity, and rhythmicity-maintaining lncRNAs.

### 3.8 Conservation of rhythmically expressed mouse testicular lncRNAs with humans, rats, and zebrafish

We performed conservation analyses of 519 rhythmically expressed mouse testicular lncRNAs from the 12-time-point dataset, as well as 475 and 494 rhythmically expressed mouse testicular lncRNAs from the six-time-point control and desynchronized datasets, with humans, rats, and zebrafish. The potential lncRNA orthologs were predicted with local BLAST with E-value E-50 threshold as a similarity measure. Out of the 519 lncRNAs from the 12-time-point dataset, we found 57 rhythmically expressed mouse lncRNAs conserved with humans (Supplementary Table 39), 259 rhythmically expressed mouse lncRNAs conserved with rats (Supplementary Table 40), while only one rhythmically expressed mouse lncRNA is conserved with zebrafish (Supplementary Table 41). Interestingly, one lncRNA (SUDAMMLNC2705) was conserved among all four species (Supplementary Table 47).

Out of the 475 lncRNAs from the six-time-point control dataset, we found 70 rhythmically expressed mouse lncRNAs conserved with humans (Supplementary Table 42), 293 rhythmically expressed mouse lncRNAs conserved with rats (Supplementary Table 43), while six rhythmically expressed mouse lncRNA are conserved with zebrafish (Supplementary Table 44). Interestingly, two lncRNAs (SUDAMMLNC237 and SUDAMMLNC1860) were conserved among all four species (Supplementary Table 48). Further, out of the 494 lncRNAs from the six-time-point desynchronized dataset, we found 75 rhythmically expressed mouse lncRNAs conserved with humans (Supplementary Table 45), 305 rhythmically expressed mouse lncRNAs conserved with rats (Supplementary Table 46), while no rhythmically expressed mouse lncRNA are conserved with zebrafish.

We used CPAT [57] to predict the peptides encoded by smORFs of the one rhythmically expressed conserved lncRNA from the 12-time-point dataset and the two conserved lncRNA from the six-time-point control dataset, and investigated their 3D models and functions. Since no lncRNAs from the desynchronized condition were conserved among all four species, we excluded their analyses for 3D models and functions. In particular, for 12-time-point wild-type dataset, we determined the peptides encoded by mouse lncRNA SUDAMMLNC2705 (NONCODE ID chr1_129801156–129803130), and its human orthologs lncRNA (NONHSAT066059.2), rat orthologs lncRNA (NONRATT008265.2), and zebrafish orthologs lncRNA (ZFLNCT09346) and analyzed the conservation of peptides to determine their 3D models and functions. We computationally identified 20 peptides encoded by smORFs of mouse lncRNA SUDAMMLNC2705 (Supplementary Table 49), four peptides encoded by human lncRNA NONHSAT066059.2 (Supplementary Table 50), two peptides encoded by rat lncRNA NONRATT008265.2 (Supplementary Table 51), and six peptides encoded by zebrafish lncRNA ZFLNCT09346 (Supplementary Table 52). We performed local BLAST to compare these peptide sequences; however, no lncRNA-encoded peptides are conserved among the four species. For the six-time-point control dataset, we determined the peptides encoded by mouse lncRNA SUDAMMLNC237, and its human orthologs lncRNA (NONHSAT228067.1), rat orthologs lncRNA (NONRATT030322.2), and zebrafish orthologs lncRNA (ZFLNCT08764), and analyzed the conservation of peptides to determine their 3D models and functions. We computationally identified 243 peptides encoded by smORFs of mouse lncRNA SUDAMMLNC237 (Supplementary Table 53), 26 peptides encoded by smORFs of human lncRNA NONHSAT228067.1 (Supplementary Table 54), 27 peptides encoded by smORFs of rat lncRNA NONRATT030322.2 (Supplementary Table 55), and 20 peptides encoded by smORFs of zebrafish lncRNA ZFLNCT08764 (Supplementary Table 56). We performed local BLAST to compare these peptide sequences; although no lncRNA-encoded peptides are conserved among the four species, three lncRNA-encoded peptides are conserved between mice and rats (Supplementary Table 57). Further, we also determined the peptides encoded by the smORF of mouse lncRNA SUDAMMLNC1860, and its human orthologs lncRNA (NONHSAT232061.1), rat orthologs lncRNA (NONRATT030711.2), and zebrafish orthologs lncRNA (ZFLNCT00095) and analyzed the conservation of peptides and their 3D models and functions. The mouse lncRNA SUDAMMLNC1860 encoded 147 peptides (Supplementary Table 58), whereas its human orthologs lncRNA NONHSAT232061.1 encoded eight peptides (Supplementary Table 59), rat orthologs lncRNA NONRATT030711.2 encoded three peptides (Supplementary Table 60), and zebrafish orthologs lncRNA ZFLNCT00095 encoded five peptides (Supplementary Table 61). We performed local BLAST to compare these peptide sequences; although no lncRNA-encoded peptides are conserved among the four species, one lncRNA-encoded peptide is conserved between mice and humans (Supplementary Table 62), whereas two lncRNA-encoded peptides are conserved between mice and rats (Supplementary Table 63).

We predicted the 3D models and functions of the lncRNA-encoded peptides with (PS)2-v2 Protein Structure Prediction Server (Fig. 8D-8E). The computational analyses found 3D model and functions in the Protein Data Bank (http://www.rcsb.org/) for three lncRNA-encoded micropeptides (Supplementary Table 64). Two mouse lncRNA-encoded peptides (SUDAMMLNC1860_131 and SUDAMMLNC1860_39) and one rat lncRNA-encoded peptides (NONRATT030711.2_3) have known functions and 3D models. The plots of the 3D models (Fig. 8D-8E) of mouse and rat lncRNA-encoded micropeptides show close similarities in terms of α-helix, β-strand, and random coils.

Subsequently, we performed the conservative analysis of the peptides encoded by these conserved lncRNAs. We performed the local BLASTs to compare the 24 mouse testicular lncRNA-encoded peptides with six human lncRNA-encoded peptides, four rat lncRNA-encoded peptides, and one zebrafish lncRNA-encoded peptide, respectively. However, no peptides are conserved among them, potentially suggesting a different operating mechanism of circadian rhythms in these four different species. Together, we identified 24, six, four, and one lncRNA-encoded peptides in mice, humans, rats, and zebrafish, plotted the available 3D models of these peptides, and performed conservative analysis at the amino acid level.

## 4 Discussion

Despite thousands of lncRNAs and their corresponding expression patterns being profiled [25], [55], much remains elusive about their involvement in regulating circadian clocks. Our previous studies [23], [64] have cataloged coding potentials of numerous lncRNAs and their encoded micropeptides, lncRNAs’ expression profiles, rhythmically expressed lncRNAs, and their potential roles in regulating circadian clocks in zebrafish [9], [31]. Additional computational and experimental studies are required to uncover the hidden patterns in the experimental profiles.

In this study, we identified a total of 1345 rhythmically expressed lncRNAs in the mouse testis. Compared to a previous study [65] that investigated approximately 100 lncRNAs in the rat pineal gland, we generated 18,613 unannotated mouse testicular transcripts measured at 12 time points under light-dark (LD) conditions and 21,414 transcripts of two six-time-point datasets under desynchronized and control conditions, respectively. We employed state-of-the-art computational tools to identify 5964 lncRNAs. The rhythmicity analysis with MetaCycle revealed 519, 475, and 494 rhythmically expressed lncRNAs in the mouse testis under 12-time-point, six-time-point control, and six-time-point desynchronized datasets, respectively. To understand the loss and gain of rhythmicity between control and desynchronized conditions and identified, we compared expression profiles of the rhythmically expressed lncRNAs and identified 427 loss-of-rhythmicity lncRNAs and 446 gain-of-rhythmicity lncRNAs in the desynchronized condition, and 48 rhythmicity-maintaining lncRNAs between control and desynchronized conditions. Further, the rhythmically expressed lncRNAs are classified into morning lncRNAs, evening lncRNAs, and night lncRNAs and investigated for their potential regulation by E-Box, D-Box, or RORE promoter motifs. The GO and KEGG pathway enrichment analyses revealed the possible involvement of these rhythmically expressed lncRNAs in numerous crucial biological processes, such as cell cycle, mitosis, cellular senescence, mitotic G1 phase, and G1/S transition.

Albeit all the promising strengths of our study in integrating experimental observation with computational tools and techniques, this study is constrained by several bioinformatic limitations inherent to computational analyses. First, we employ the RNA-seq expression profiles to investigate the rhythmically expressed mouse testicular lncRNAs. However, RNA-seq datasets have their own limitations [66]. For example, the RNA-seq technology does not allow for identifying those lncRNAs without poly(A) tails [67]. Second, several lncRNA-encoded peptides identified (Supplementary Tables 49–56, and 58–61) are longer than 100 amino acids. Although our research framework can be used to investigate canonical peptides, as well as the micropeptides arbitrarily defined as containing up to 100 amino acids [28], additional studies are required to exclusively investigate the lncRNA-encoded micropeptides. Third, the FIMO utility only allows for a few choices of *P*-values for predicting E-Box, D-Box, or RORE motifs in promoter regions. Hence, the tool is prone to report false positives. Fourth, despite this study identifying thousands of rhythmically expressed mouse testicular lncRNAs, it’s likely that there are more lncRNAs involved in circadian regulation in different mouse tissues. Distinct testicular cell types may also affect the circadian, and additional experiments are required to understand such regulatory mechanisms. Due to the limitation of the availability of time-series data of different mouse tissues, our study primarily focused on mouse testis. As more relevant data become available in the future, it will be interesting to examine how many of these rhythmically expressed lncRNAs are co-expressed in different mouse tissues. Fifth, our data were profiled under LD conditions. Comparing the rhythmically expressed lncRNAs in LD conditions with other conditions, such as constant darkness (DD) and constant light (LL) remains an open research direction. Sixth, despite the 12-time-point dataset and six-point control dataset sharing the same number of 5964 lncRNAs, we identified 44 more rhythmically expressed in the 12-time-point dataset than in the six-point control dataset. As differing sampling strategies [62], [63] can affect the expression profiles, additional studies are required to validate the effects of sampling on the outcomes of the rhythmicity analysis. Seventh, although the computational tools helped predict GO and KEGG pathway annotations of these rhythmically expressed mouse testicular lncRNAs, these predictions require experimental validations. Finally, although our data is derived directly from biological experiments, additional experimental validation is required to confirm the computational predictions presented in this study. Despite all the bioinformatic limitations unavoidable to computational studies, our study brings numerous interesting findings. To the best of our knowledge, this is the first time that thousands of rhythmically expressed lncRNAs have been identified in the mouse testis. Our study framework integrating experimental data with computational analyses can be adapted to investigate a large number of lncRNAs from a diverse set of organisms. The study should help select the lncRNAs of interest prior to performing time-intensive biological experiments.

## 5 Conclusion

Our integrative framework of experimental observations and state-of-the-art bioinformatic tools and techniques revealed thousands of oscillating lncRNAs in the mouse testis. As more experimental data and computation tools become available in the future, our proposed framework from this study can be used to elucidate the interplay of lncRNAs and circadian clocks.

## CRediT authorship contribution statement

SM and HW conceptualized the research design, investigation, and methodology. SM performed the literature review, data curation, computational analysis, writing–original draft, and formal analysis. TL performed experiments to generate the time-course data, LimoRhyde analyses and gave feedback to improve the manuscript. HW supervised the whole project, acquired funding, reviewed, and edited the manuscript. All authors agreed and accepted the final version of the manuscript before submission.

## Declaration of Competing Interest

The authors have declared that no competing interest exists.
