# Supplementary material for: Thousands of oscillating LncRNAs in the mouse testis
Source: Comput Struct Biotechnol J. 2023 Nov 29;23:330–46. doi: 10.1016/j.csbj.2023.11.046 (PMC10776378; doi:10.1016/j.csbj.2023.11.046)
Supplement: Supplementary file 1 — Supplementary material [file mmc1.docx]

Supplementary Materials for

Thousands of Oscillating LncRNAs in the Mouse Testis

Shital Kumar Mishra^1,2‡^, Taole Liu^1,2‡^, Han Wang^1,2^*

1. Center for Circadian Clocks, Soochow University, Suzhou 215123, Jiangsu, China

2. School of Biology & Basic Medical Sciences, Suzhou Medical College, Soochow University, Suzhou 215123, Jiangsu, China

^‡^ These authors contributed equally to the work.

* Correspondence: han.wang88@gmail.com, wanghan@suda.edu.cn; Tel.: +86 51265882115

**This PDF file includes**:

Titles of Supplementary Tables S1 to S64

Supplementary Figures S1 to S23

**Other supplementary materials for this manuscript include the following:**

Supplementary Tables S1 to S64

**Titles of Supplementary Tables S1 to S64**

**Supplementary Table 1**. Expression profiles of mouse testis transcripts from the 12-time-point dataset.

**Supplementary Table 2**. Expression profiles of mouse testis transcripts from the six-time-point control dataset.

**Supplementary Table 3**. Expression profiles of mouse testis transcripts from the six-time-point desynchronized dataset.

**Supplementary Table 4**. Averaged expression profiles of mouse testicular lncRNAs in the 12-time-point dataset and their rhythmicity analyses with MetaCycle.

**Supplementary Table 5**. Averaged expression profiles of mouse testicular lncRNAs in the six-time-point control dataset and their rhythmicity analyses with MetaCycle.

**Supplementary Table 6**. Averaged expression profiles of mouse testicular lncRNAs in the six-time-point desynchronized dataset and their rhythmicity analyses with MetaCycle.

**Supplementary Table 7**. Rhythmically expressed mouse testicular lncRNAs from the 12-time-point dataset grouped into morning lncRNAs, evening lncRNAs, and night lncRNAs.

**Supplementary Table 8**. Rhythmically expressed mouse testicular lncRNAs from the six-time-point control dataset grouped into morning lncRNAs, evening lncRNAs, and night lncRNAs.

**Supplementary Table 9**. Rhythmically expressed mouse testicular lncRNAs from the six-time-point desynchronized dataset grouped into morning lncRNAs, evening lncRNAs, and night lncRNAs.

**Supplementary Table 10**. Ensemble IDs of rhythmically expressed morning lncRNAs, evening lncRNAs, and night lncRNAs from the 12-time-point dataset.

**Supplementary Table 11**. Ensemble IDs of rhythmically expressed morning lncRNAs, evening lncRNAs, and night lncRNAs from the six-time-point control dataset.

**Supplementary Table 12**. Ensemble IDs of rhythmically expressed morning lncRNAs, evening lncRNAs, and night lncRNAs from the six-time-point desynchronized dataset.

**Supplementary Table 13**. Promoter analysis of morning lncRNAs from the 12-time-point dataset with FIMO.

**Supplementary Table 14**. Promoter analysis of evening lncRNAs from the 12-time-point dataset with FIMO.

**Supplementary Table 15**. Promoter analysis of night lncRNAs from the 12-time-point dataset with FIMO.

**Supplementary Table 16**. GO annotations of the morning mouse testicular lncRNAs from the 12-time-point dataset.

**Supplementary Table 17**. GO annotations of the evening mouse testicular lncRNAs from the 12-time-point dataset.

**Supplementary Table 18**. GO annotations of the evening mouse testicular lncRNAs from the 12-time-point-dataset.

**Supplementary Table 19**. Promoter analysis of morning lncRNAs from the six-time-point control dataset with FIMO.

**Supplementary Table 20**. Promoter analysis of evening lncRNAs from the six-time-point FIMO.

**Supplementary Table 21**. Promoter analysis of night lncRNAs from the six-time-point control dataset FIMO.

**Supplementary Table 22**. GO annotations of the morning mouse testicular lncRNAs from the six-time-point control dataset.

**Supplementary Table 23**. GO annotations of the evening mouse testicular lncRNAs from the six-time-point control dataset.

**Supplementary Table 24**. GO annotations of the evening mouse testicular lncRNAs from the six-time-point control dataset.

**Supplementary Table 25**. **46** rhythmically expressed mouse testicular lncRNAs shared between the 12-time-point dataset and the six-time-point control dataset with corresponding data from the 12-time-point dataset and their classification into the morning lncRNAs, evening lncRNAs, and the night lncRNAs.

**Supplementary Table 26**. **46** rhythmically expressed mouse testicular lncRNAs shared between the 12-time-point dataset and the six-time-point control dataset with corresponding data from the six-time-point dataset and their classification into the morning lncRNAs, evening lncRNAs, and the night lncRNAs.

**Supplementary Table 27**. Promoter analysis of morning lncRNAs from the six-time-point desynchronized dataset with FIMO.

**Supplementary Table 28**. Promoter analysis of evening lncRNAs from the six-time-point desynchronized dataset with FIMO.

**Supplementary Table 29**. Promoter analysis of night lncRNAs from the six-time-point desynchronized dataset with FIMO.

**Supplementary Table 30S**. GO annotations of the morning mouse testicular lncRNAs from the six-time-point desynchronized dataset.

**Supplementary Table 31**. GO annotations of the evening mouse testicular lncRNAs from the six-time-point desynchronized dataset.

**Supplementary Table 32**. GO annotations of the night mouse testicular lncRNAs from the six-time-point desynchronized dataset.

**Supplementary Table 33**. ENSEMBLIDs t of loss-of-rhythmicity lncRNAs and gain-of-rhythmicity lncRNAs in the desynchronized condition, and rhythmicity-maintaining lncRNAs between control and desynchronized conditions.

**Supplementary Table 34**. Expression patterns of loss-of-rhythmicity lncRNAs in desynchronized condition and their classification into morning lncRNAs, evening lncRNAs, and night lncRNAs using data from the control condition.

**Supplementary Table 35**. Expression patterns of loss-of-rhythmicity lncRNAs in the desynchronized condition and their classification into morning lncRNAs, evening lncRNAs, and night lncRNAs using data from desynchronized condition.

**Supplementary Table 36**. Expression patterns of rhythmicity-maintaining lncRNAs between control and desynchronized conditions and their classification into morning lncRNAs, evening lncRNAs, and night lncRNAs using data from the control condition.

**Supplementary Table 37**. Expression patterns of rhythmicity-maintaining lncRNAs between control and desynchronized conditions and their classification into morning lncRNAs, evening lncRNAs, and night lncRNAs using data from the desynchronized condition.

**Supplementary Table 38**. MetaCycle *P*-values of the representative lncRANs from the control and desynchronized datasets that lost, gained, and maintained rhythmicity between control and desynchronized conditions.

**Supplementary Table 39**. Rhythmically expressed mouse testicular lncRNAs from the 12-time-point dataset conserved with humans’.

**Supplementary Table 40**. Rhythmically expressed mouse testicular lncRNAs from the 12-time-point dataset conserved with rats’.

**Supplementary Table 41**. Rhythmically expressed mouse testicular lncRNAs from the 12-time-point dataset conserved with zebrafish’s.

**Supplementary Table 42**. Rhythmically expressed mouse testicular lncRNAs from the six-time-point control dataset conserved with humans’.

**Supplementary Table 43**. Rhythmically expressed mouse testicular lncRNAs from the six-time-point control dataset conserved with rats’.

**Supplementary Table 44**. Rhythmically expressed mouse testicular lncRNAs from the six-time-point control dataset conserved with zebrafish’s.

**Supplementary Table 45**. Rhythmically expressed mouse testicular lncRNAs from the six-time-point desynchronized dataset conserved with humans’.

**Supplementary Table 46**. Rhythmically expressed mouse testicular lncRNAs from the six-time-point desynchronized dataset conserved with rats’.

**Supplementary Table 47**. Rhythmically expressed mouse testicular lncRNAs from the 12-time-point dataset conserved with all four species humans, mice, rats, and zebrafish.

**Supplementary Table 48**. Rhythmically expressed mouse testicular lncRNAs from the six-time-point control dataset conserved with all four species humans, mice, rats, and zebrafish.

**Supplementary Table 49**. Mouse lncRNA SUDAZFLNC2705 encoded peptides.

**Supplementary Table 50**. Human lncRNA NONHSAT066059.2 encoded peptides.

**Supplementary Table 51**. Rat lncRNA NONRATT008265.2 encoded peptides.

**Supplementary Table 52**. Zebrafish lncRNA ZFLNCT09346 encoded peptides.

**Supplementary Table 53**. Mouse lncRNA SUDAZFLNC237 encoded peptides.

**Supplementary Table 54**. Human lncRNA NONHSAT228067.1 encoded peptides.

**Supplementary Table 55**. Rat lncRNA NONRATT030322.2 encoded peptides.

**Supplementary Table 56**. Zebrafish lncRNA ZFLNCT08764 encoded peptides.

**Supplementary Table 57**. Mouse lncRNA SUDAZFLNC237 encoded peptide conserved with rat.

**Supplementary Table 58**. Mouse lncRNA SUDAZFLNC1860 encoded peptides.

**Supplementary Table 59**. Human lncRNA NONHSAT232061.1 encoded peptides.

**Supplementary Table 60**. Rat lncRNA NONRATT030711.2 encoded peptides.

**Supplementary Table 61**. Zebrafish lncRNA ZFLNCT00095 encoded peptides.

**Supplementary Table 62**. Mouse lncRNA SUDAZFLNC1860 encoded peptide conserved with human.

**Supplementary Table 63**. Mouse lncRNA SUDAZFLNC1860 encoded peptide conserved with rat.

**Supplementary Table 64**. Known domains in Protein Data Bank shared by conserved mouse testicular lncRNA-encoded peptides.

**Supplementary figures**


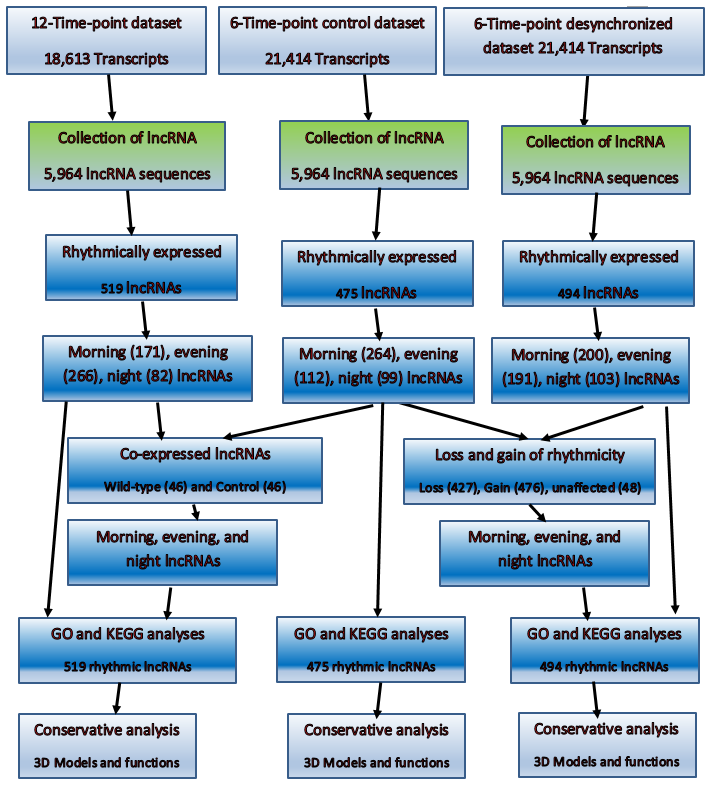


**Figure S1.** Flow diagram of the study.


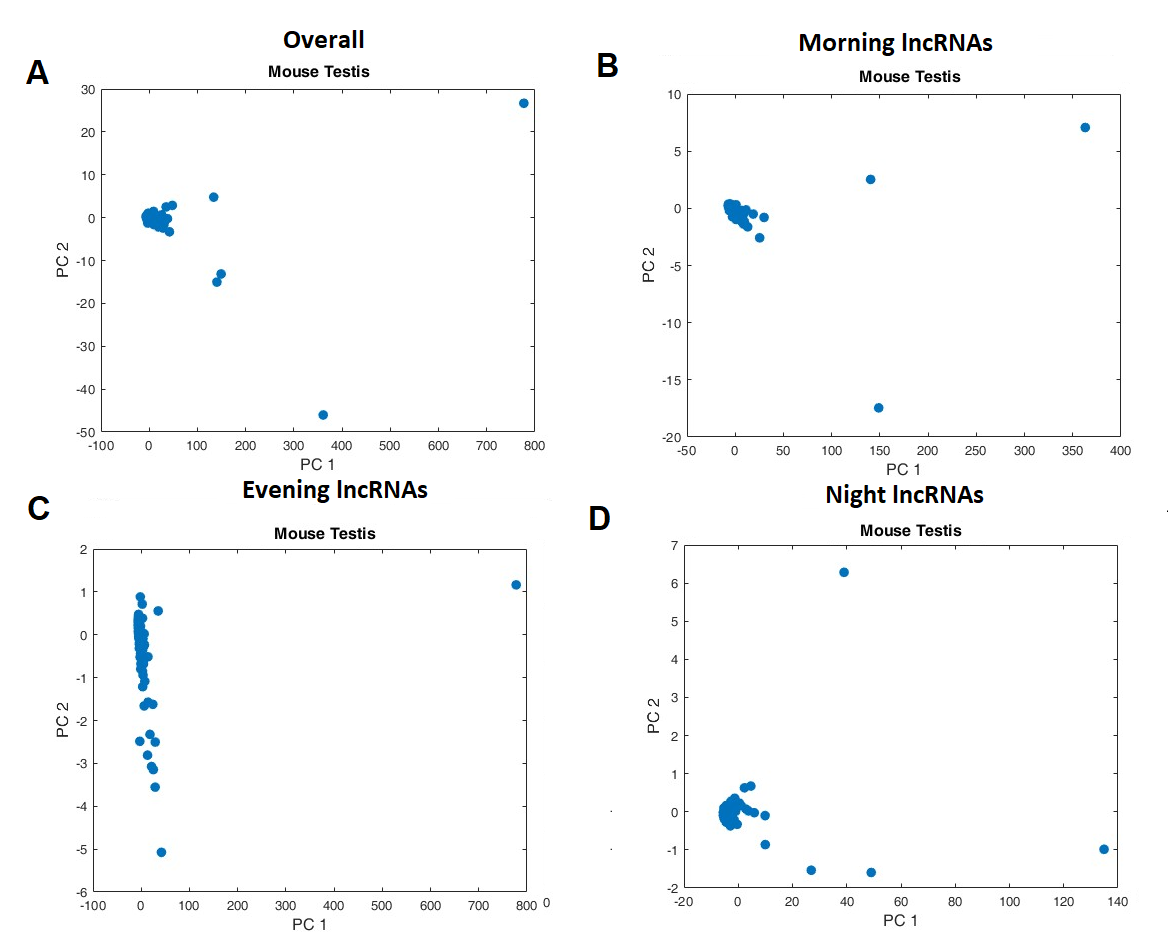


**Figure S2.** Principal Component Analysis (PCA) of rhythmically expressed mouse testicular lncRNAs in the 12-time-point dataset. PCA plots of all the 519 rhythmically expressed lncRNAs (A), 171 morning lncRNAs (B), 266 evening lncRNAs (C), and 82 night lncRNAs (D).

**
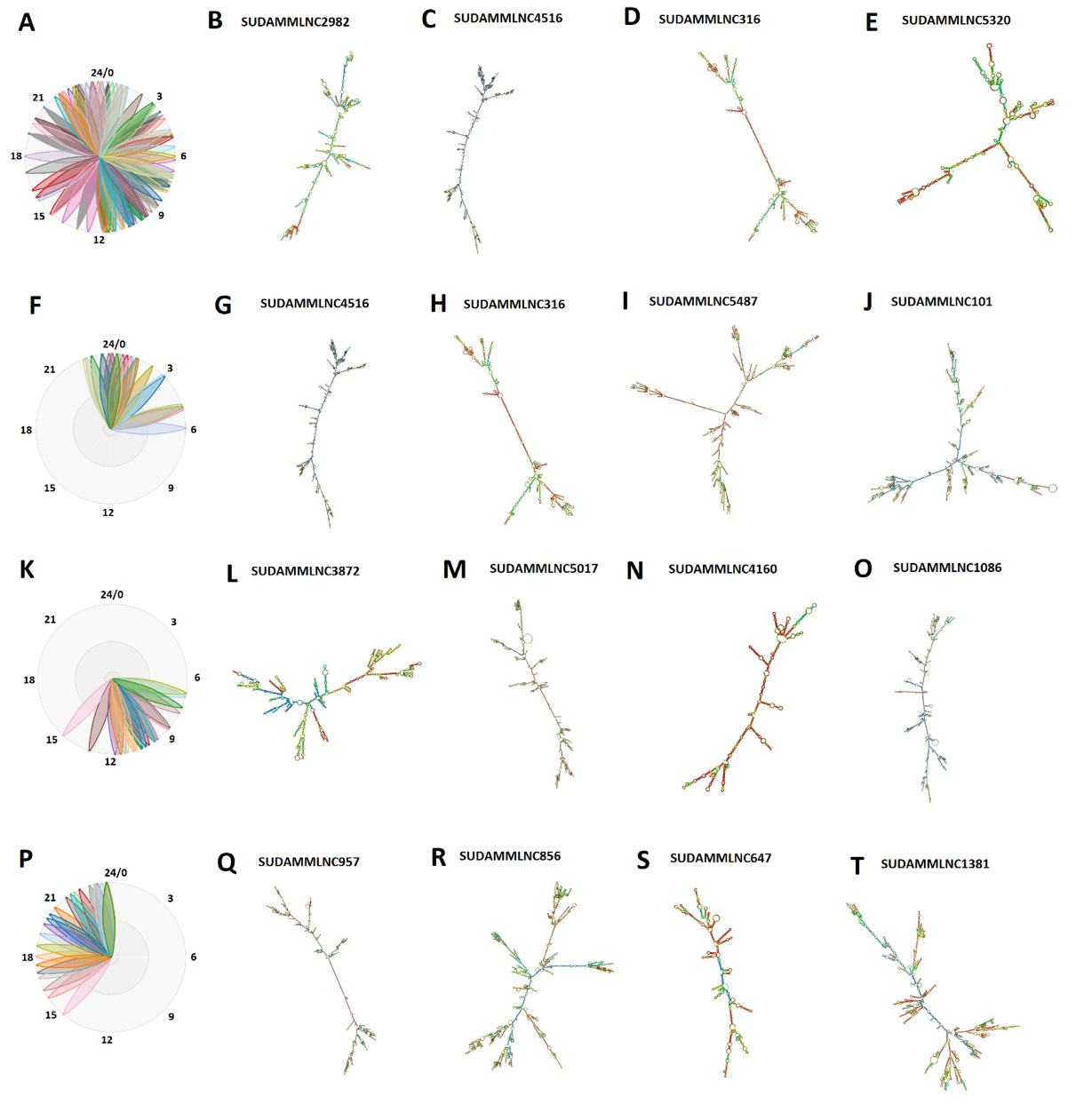
**

**Figure S3.** Analyses of expression profiles of rhythmically expressed mouse testicular lncRNAs in the 12-time-point wild-type dataset. (A-E) Analysis of all the 519 rhythmically expressed mouse testicular lncRNAs: BioDare2 plot (A) of all the 519 rhythmically expressed lncRNAs, and 2D structures of the representative lncRNAs (B-E). (F-J) Analysis of 171 morning lncRNAs: BioDare2 plot (F) of all the 171 rhythmically expressed morning lncRNAs, and 2D structures of the representative lncRNAs (G-J). (K-O) Analysis of 266 evening lncRNAs: BioDare2 plot (K) of 266 rhythmically expressed evening lncRNAs, and 2D structures of the representative lncRNAs (L-O). (P-T) Analysis of 82 night lncRNAs: BioDare2 plot (P) of 82 rhythmically expressed night lncRNAs, and 2D structures of the representative lncRNAs (Q-T).


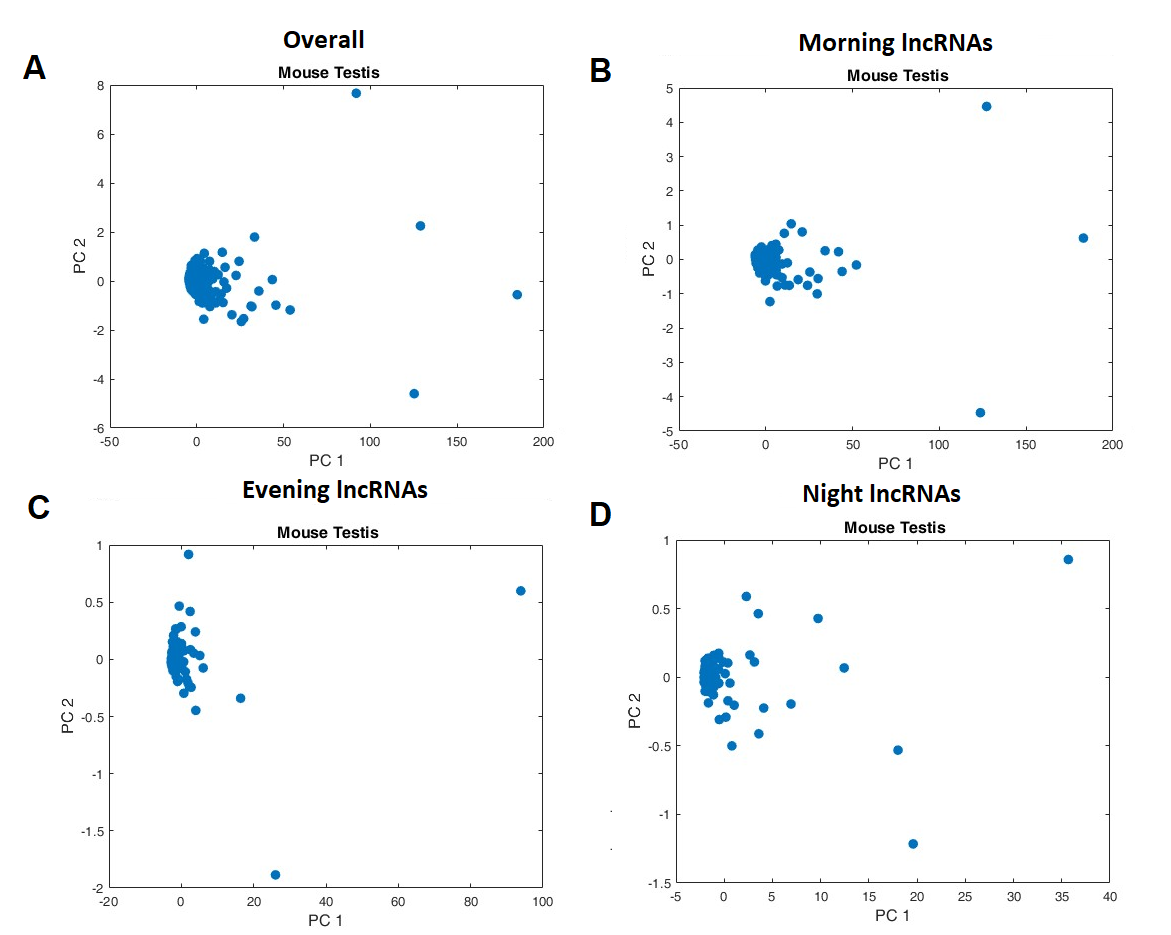


**Figure S4.** Principal Component Analysis (PCA) of 475 rhythmically expressed mouse testicular lncRNAs in the six-time-point control dataset. PCA plots of all the 475 rhythmically expressed lncRNAs (A), 264 morning lncRNAs (B), 112 evening lncRNAs (C), and 99 night lncRNAs (D).

**
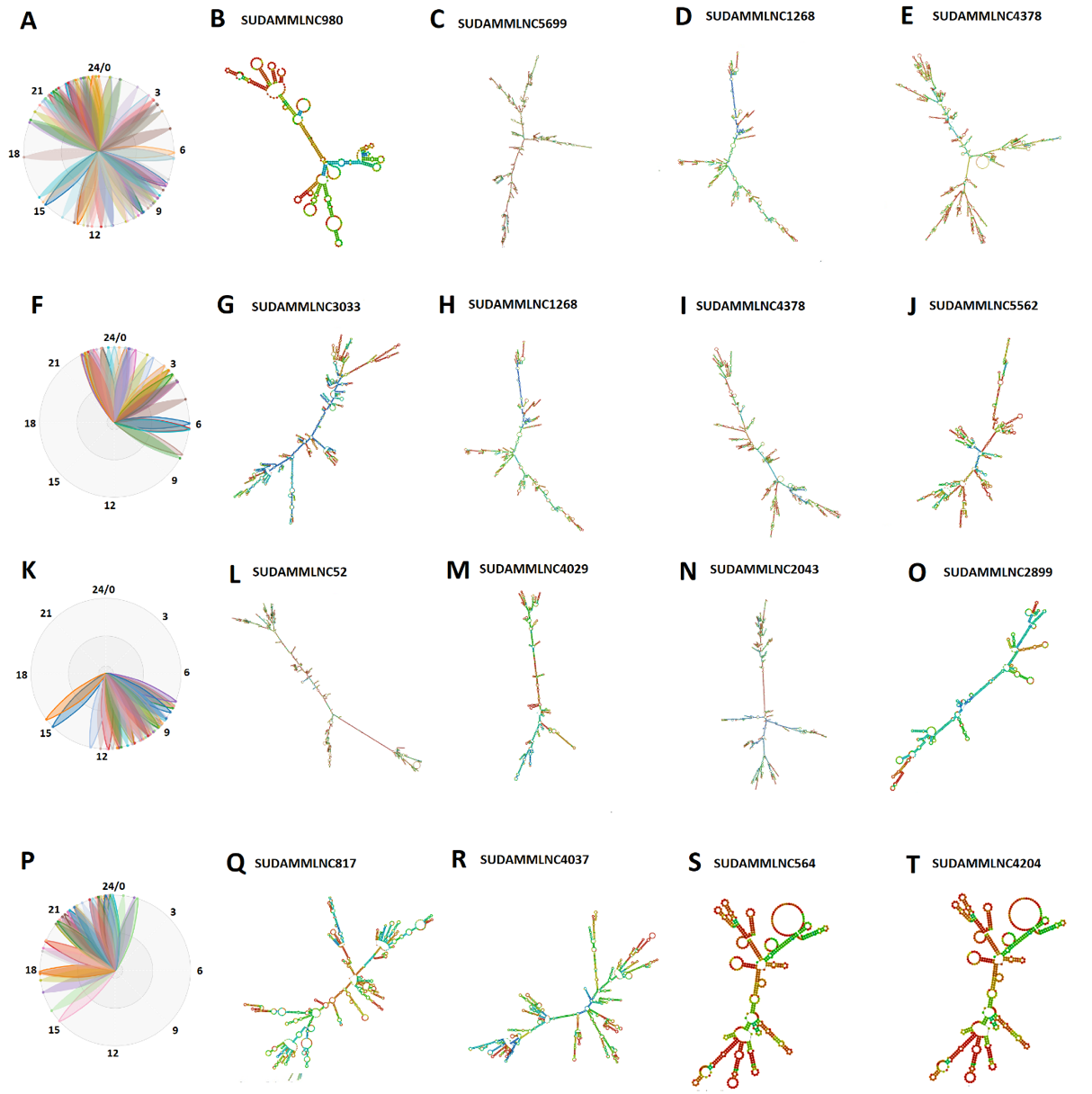
**

**Figure S5.** Analyses of expression profiles of 475 rhythmically expressed mouse testicular lncRNAs in the six-time-point control dataset. (A-E) Analysis of all the 475 rhythmically expressed mouse testicular lncRNAs: BioDare2 plot (A) of all the 475 rhythmically expressed lncRNAs, and 2D structures of the representative lncRNAs (B-E). (F-J) Analysis of 264 morning lncRNAs: BioDare2 plot (F) of all the 264 rhythmically expressed morning lncRNAs, and 2D structures of the representative lncRNAs (G-J). (K-O) Analysis of 112 evening lncRNAs: BioDare2 plot (K) of 112 rhythmically expressed evening lncRNAs, and 2D structures of the representative lncRNAs (L-O). (P-T) Analysis of 99 night lncRNAs: BioDare2 plot (P) of 99 rhythmically expressed evening lncRNAs, and 2D structures of the representative lncRNAs (Q-T).

**
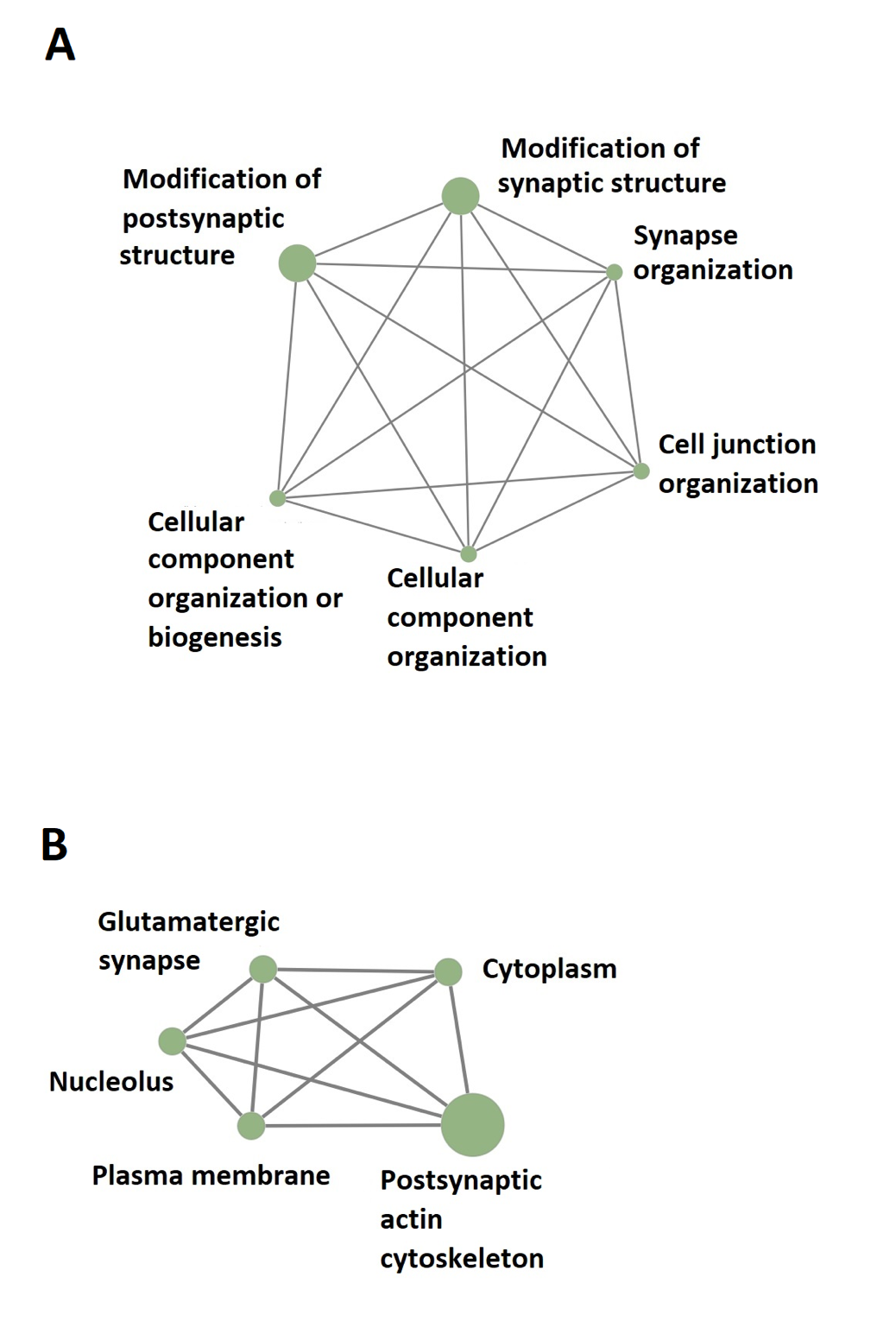
**

**Figure S6.** GO and KEGG analyses of 475 rhythmically expressed mouse testicular lncRNAs in the six-time-point control dataset measured over one day . (A) GO analysis of the lncRNAs revealed their possible involvement in numerous biological processes, such as, Modification of synaptic structure, Modification of postsynaptic structure, Synapse organization, Cell junction organization, and Cellular component organization. (B) KEGG enrichment analysis their potential biological functions, such as their involvement in Plasma membrane, Postsynaptic actin cytoskeleton, and Glutamatergic synapse.

**
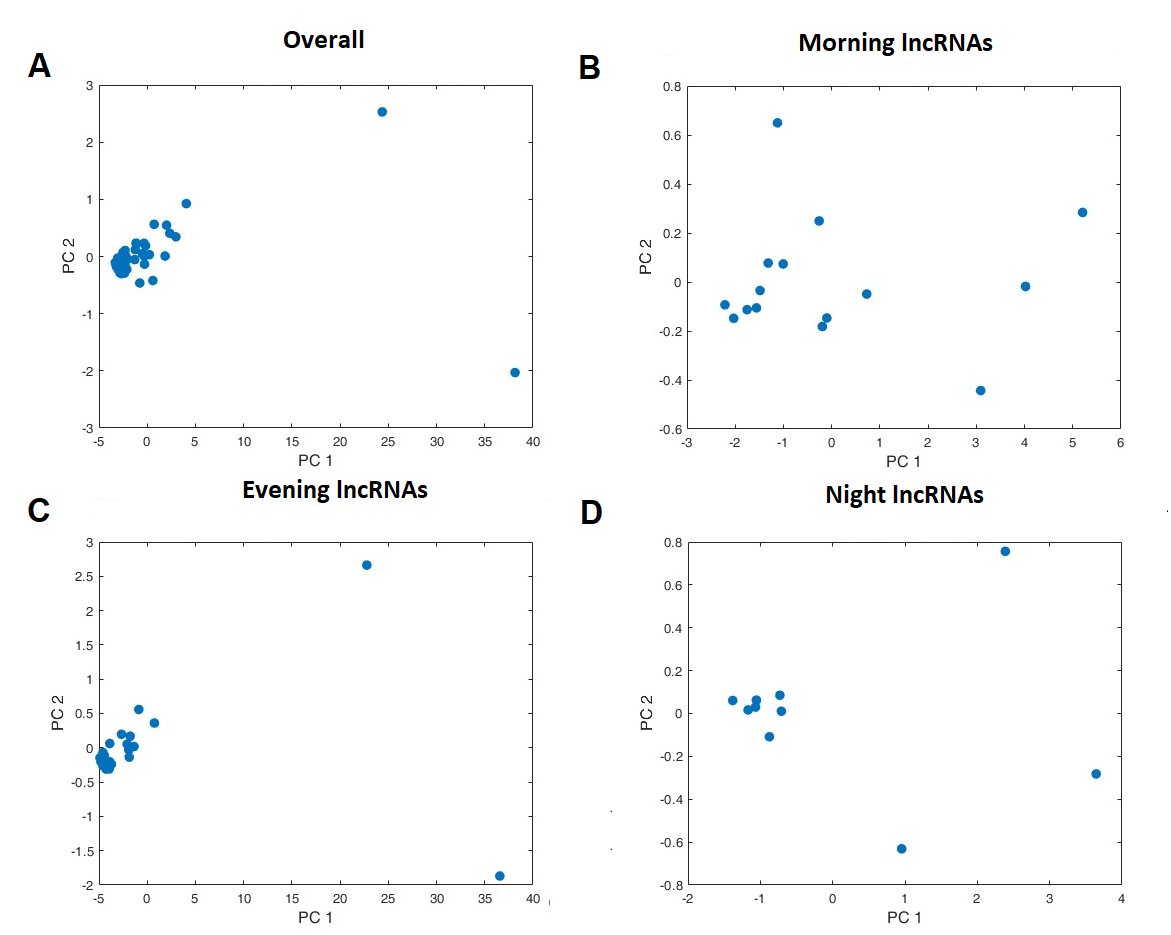
**

**Figure S7.** Principal Component Analysis (PCA) of the 46 rhythmically expressed mouse testicular lncRNAs shared between the 12-time-point dataset and six-time-point control dataset with rhythmically expressed data from the 12-time-point dataset. PCA plots of all 48 rhythmically expressed lncRNAs (A), 15 morning lncRNAs (B), 21 evening lncRNAs (C), and 10 night lncRNAs (D).


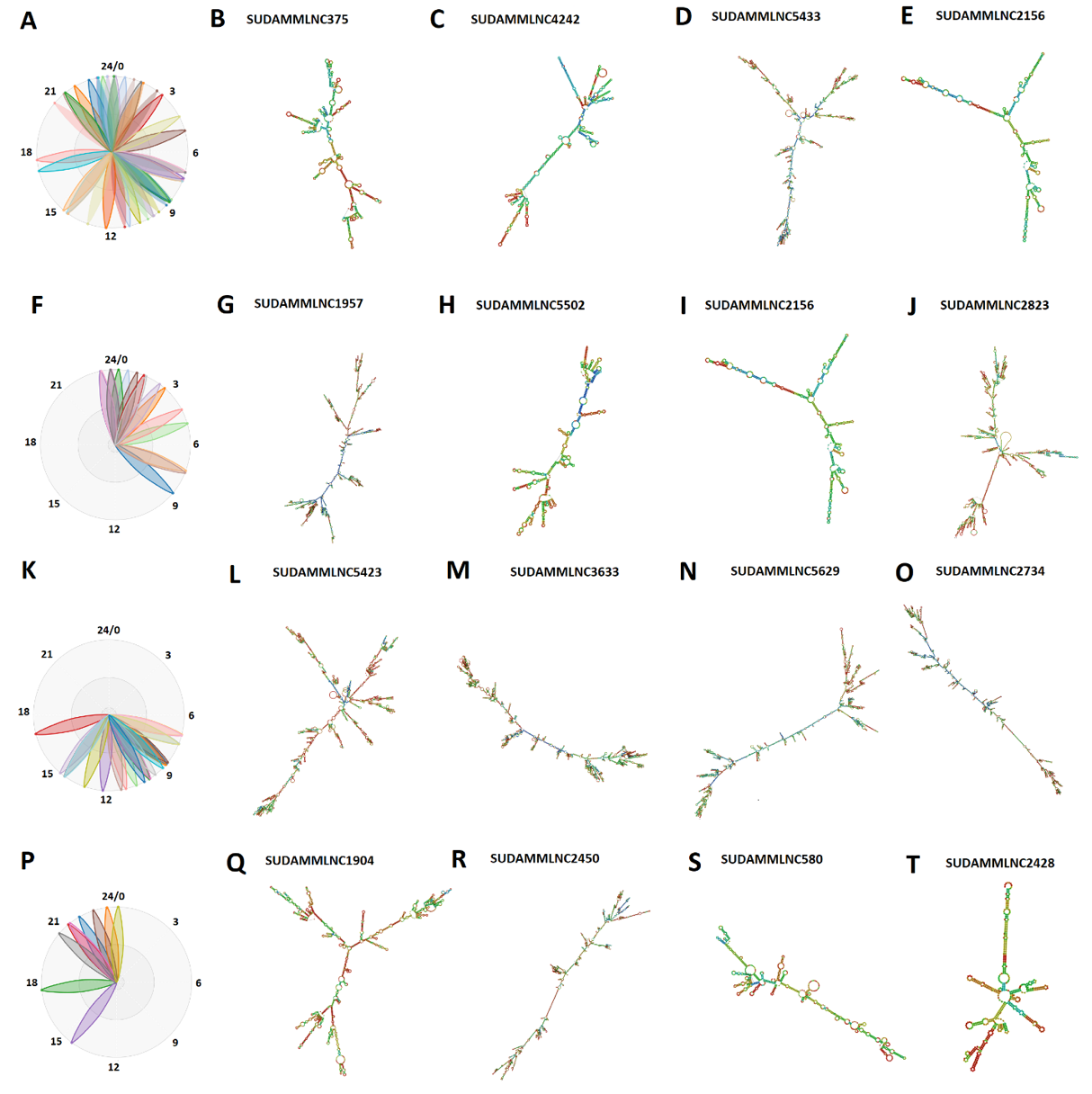


**Figure S8.** Analyses of expression profiles of the 46 rhythmically expressed mouse testicular lncRNAs shared between the 12-time-point wild-type dataset and the six-time-point control dataset with rhythmically expressed data from the 12-time-point dataset. (A-E) Analysis of all the 46 rhythmically expressed mouse testicular lncRNAs: BioDare2 plot (A) of all the 46 rhythmically expressed lncRNAs, and 2D structures of the representative lncRNAs (B-E). (F-J) Analysis of 15 morning lncRNAs: BioDare2 plot (F) of all the 15 rhythmically expressed morning lncRNAs, and 2D structures of the representative lncRNAs (G-J). (K-O) Analysis of 21 evening lncRNAs: BioDare2 plot (K) of 21 rhythmically expressed evening lncRNAs, and 2D structures of the representative lncRNAs (L-O). (P-T) Analysis of 10 night lncRNAs: BioDare2 plot (P) of 10 rhythmically expressed evening lncRNAs, and 2D structures of the representative lncRNAs (Q-T).

**
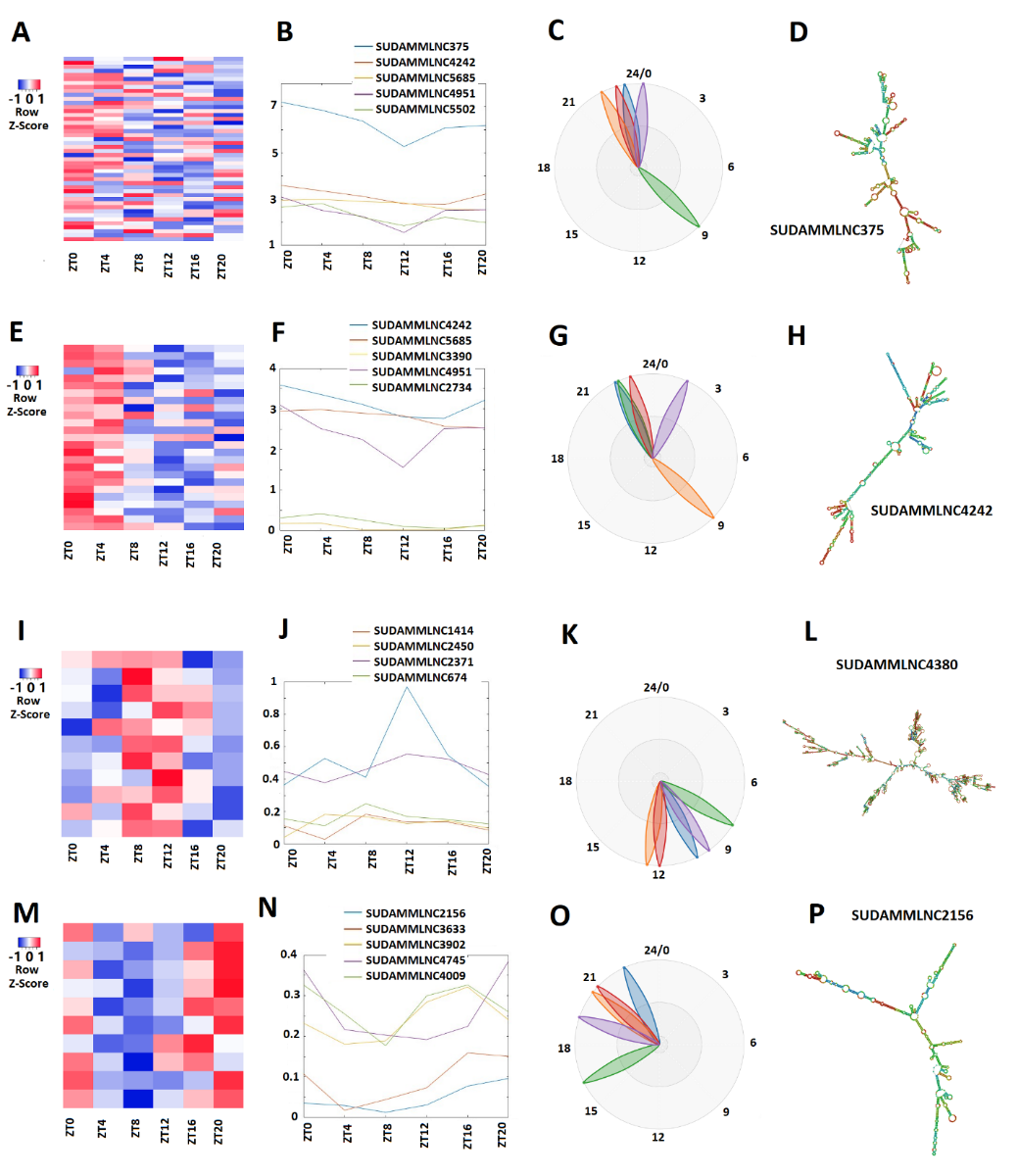
**

**Figure S9.** Analysis of 46 rhythmically expressed mouse testicular lncRNAs shared between the 12-point wild-type and 6-point control datasets with data from six-point control dataset. (A-D) Analysis of all the 46 rhythmically expressed mouse testicular lncRNAs: Heat map (A) of all the 46 rhythmically expressed lncRNAs, expression profiles (B) and phases (C) of representative lncRNAs (B), and secondary structure plot of a representative lncRNA (D). (E-H) Analysis of 25 morning lncRNAs: Heat map of the 25 morning lncRNAs (E), expression profiles (F) and phases (G) of representative morning lncRNAs (F), and secondary structure plot of a representative lncRNA (H). (I-L) Analysis of 11 evening lncRNAs: Heat map of the 11 evening lncRNAs (I), expression profiles (J) and phases (K) of representative evening lncRNAs (J), and secondary structure plot of a representative evening lncRNA (L). (M-P) Analysis of 10 night lncRNAs: Heat map of the 10 night lncRNAs (M), expression profiles (N) and phases (O), of representative evening lncRNAs (N), and secondary structure plot of a representative night lncRNA (P).


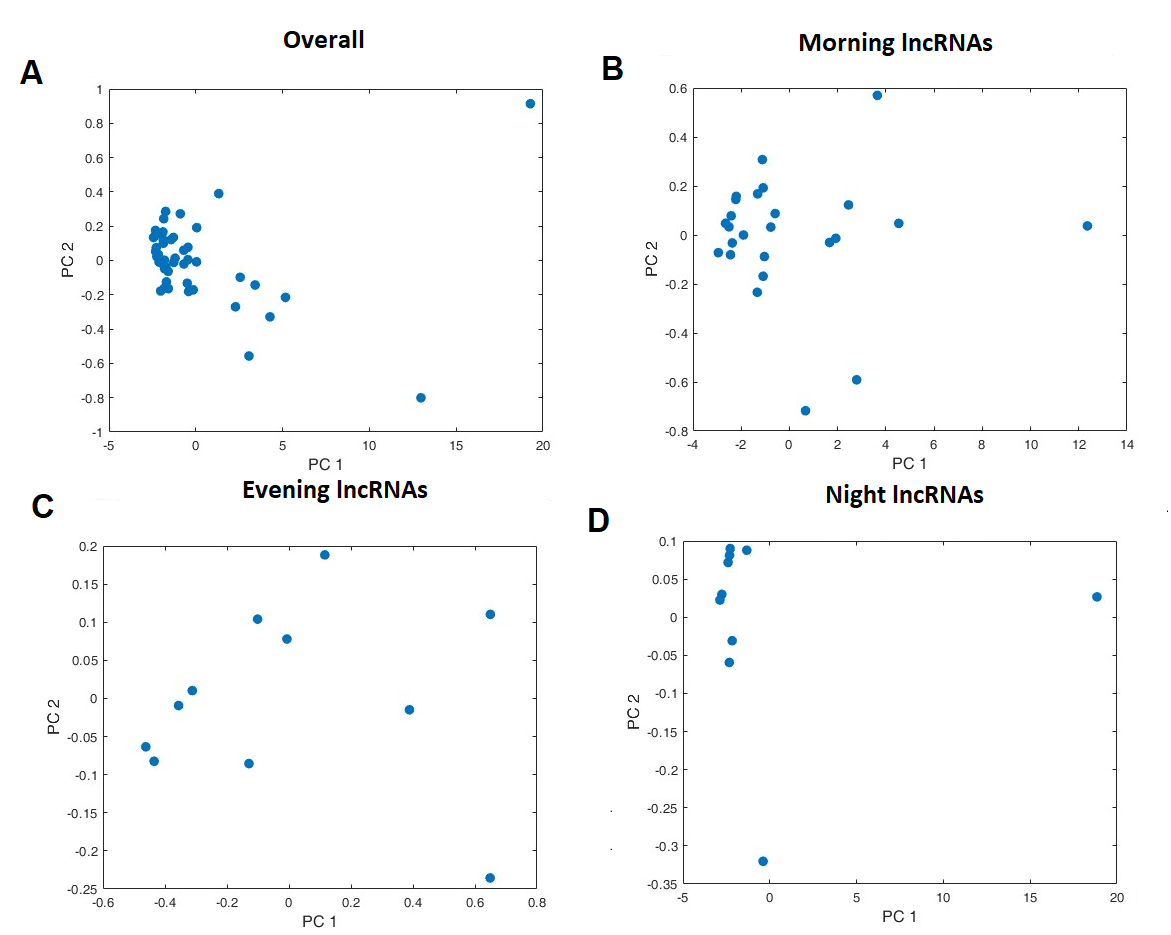


**Figure S10.** Principal Component Analysis (PCA) of the 46 rhythmically expressed mouse testicular shared between the 12-time-point dataset and six-time-point control dataset with rhythmically expressed data from the six-time-point dataset. PCA plots of all the 46 rhythmically expressed lncRNAs (A), 25 morning lncRNAs (B), 11 evening lncRNAs (C), and 10 night lncRNAs (D).


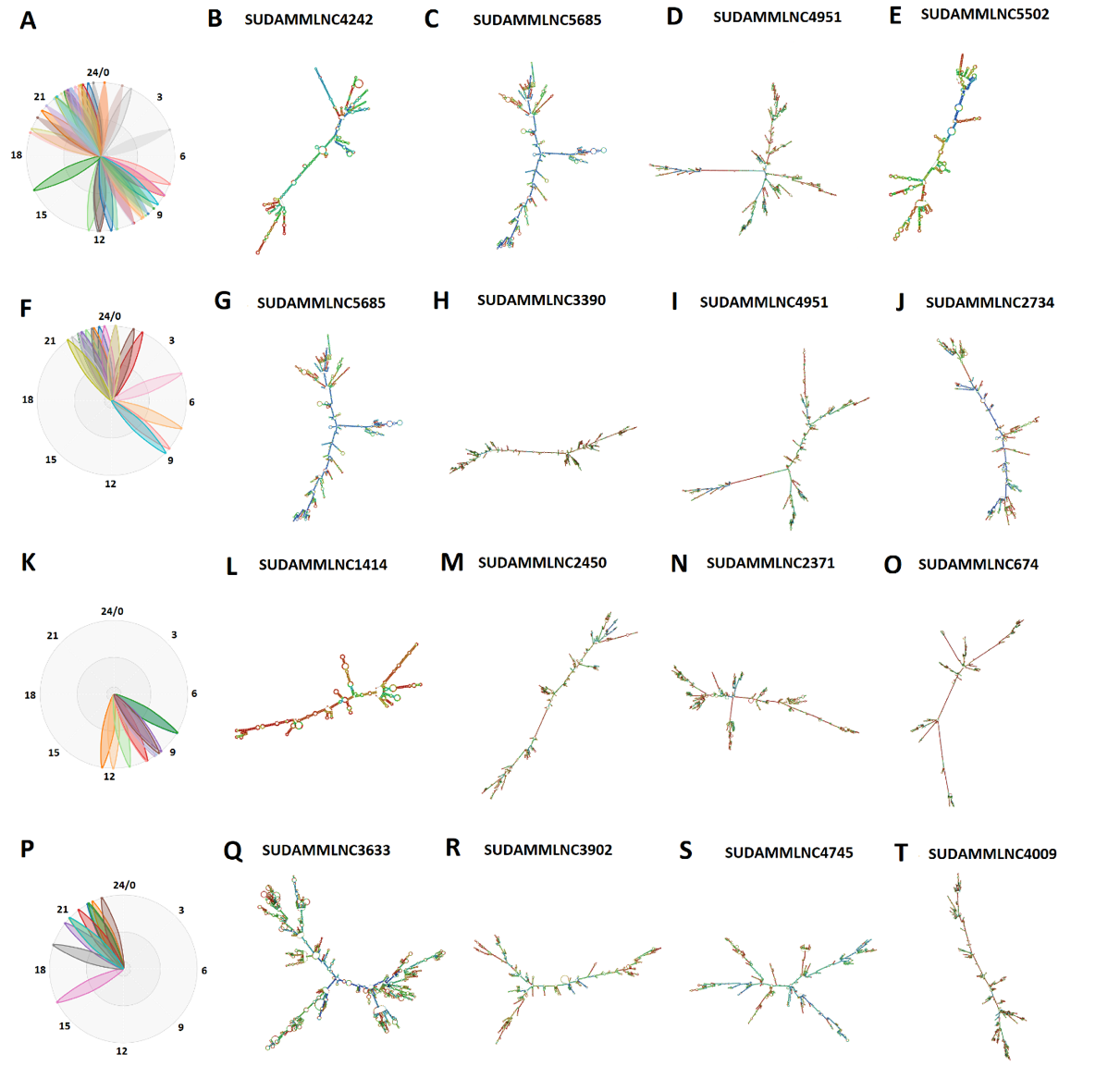


**Figure S11.** Analyses of expression profiles of 46 rhythmically expressed mouse testicular lncRNAs shared between the 12-point wild-type and six-point control datasets with rhythmically expressed data from the six-time-point dataset. (A-E) Analysis of all the 46 rhythmically expressed mouse testicular lncRNAs: BioDare2 plot (A) of all the 46 rhythmically expressed lncRNAs, and 2D structures of the representative lncRNAs (B-E). (F-J) Analysis of 25 morning lncRNAs: BioDare2 plot (F) of all the 25 rhythmically expressed morning lncRNAs, and 2D structures of the representative lncRNAs (G-J). (K-O) Analysis of 11 evening lncRNAs: BioDare2 plot (K) of 11 rhythmically expressed evening lncRNAs, and 2D structures of the representative lncRNAs (L-O). (P-T) Analysis of 10 night lncRNAs: BioDare2 plot (P) of 10 rhythmically expressed evening lncRNAs, and 2D structures of the representative lncRNAs (Q-T).


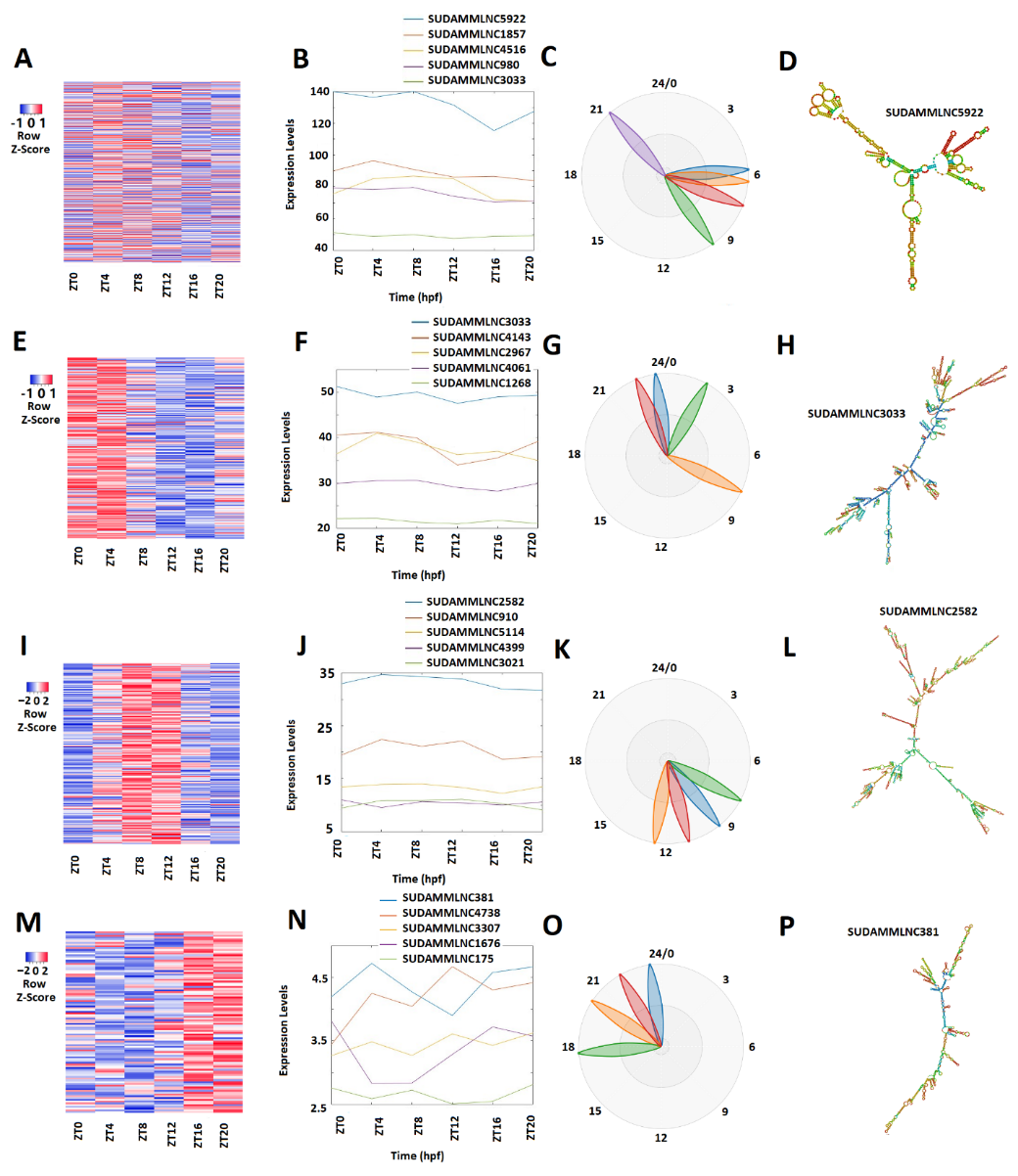


**Figure S12.** Analysis of 494 rhythmically expressed mouse testicular lncRNAs in the desynchronized dataset. (A-D) Analysis of all the 494 rhythmically expressed mouse testicular lncRNAs from the six-time-point desynchronized dataset: Heat map (A) of all the 494 rhythmically expressed lncRNAs, expression profiles (B) and phases (C) of representative lncRNAs, and secondary structure plot of a representative lncRNA (D). (E-H) Analysis of 200 morning lncRNAs: Heat map of the 200 morning lncRNAs (E), expression profiles (F) and phases (G) of representative morning lncRNAs (F), and secondary structure plot of a representative lncRNA (H). (I-L) Analysis of 191 evening lncRNAs: Heat map of the 191 evening lncRNAs (I), expression profiles (J) and phases (K) of representative evening lncRNAs (J), and secondary structure plot of a representative evening lncRNA (L). (M-P) Analysis of 103 night lncRNAs: Heat map of the 103 night lncRNAs (M), expression profiles (N) and phases (O) of representative evening lncRNAs, and secondary structure plot of a representative night lncRNA (P).

**
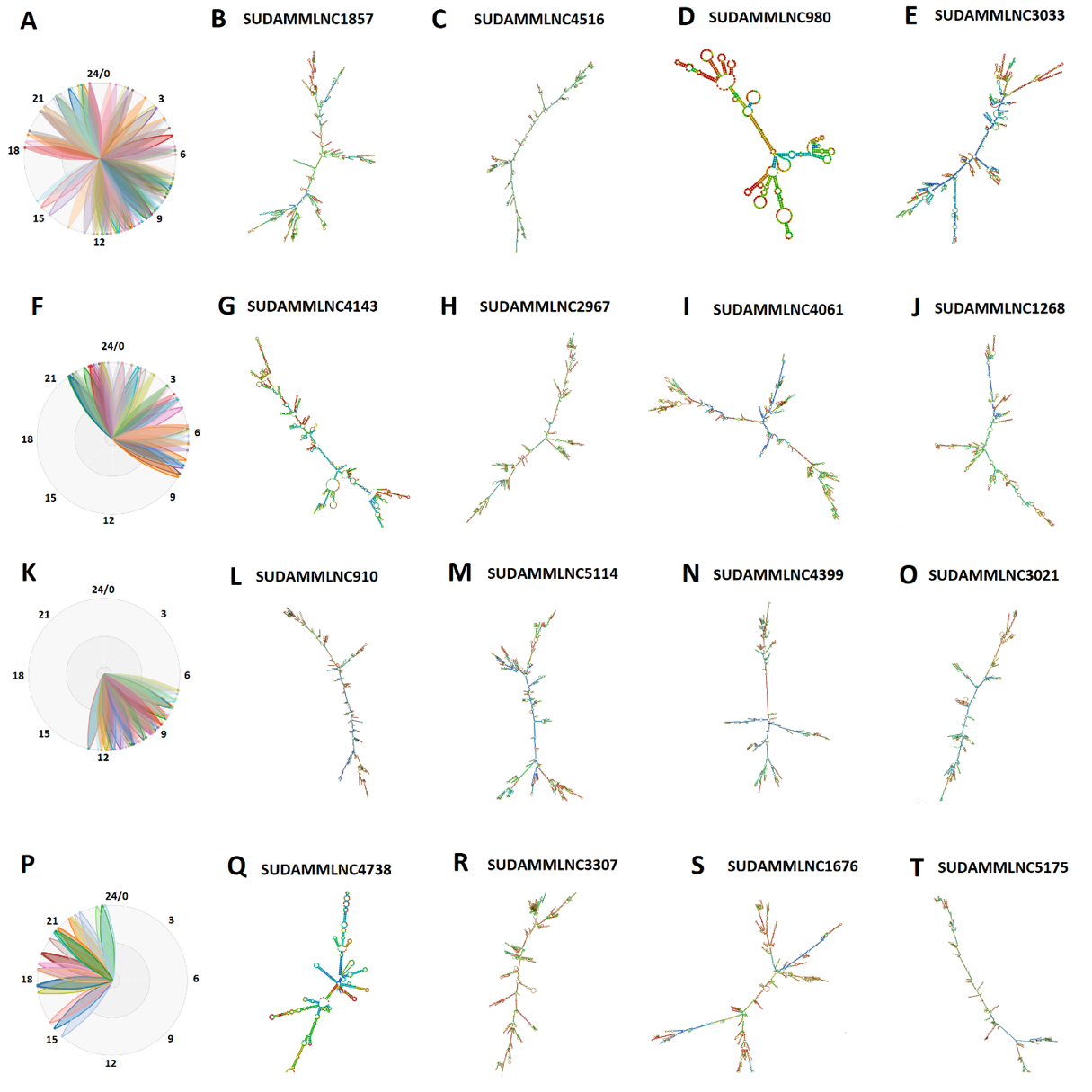
**

**Figure S13.** Analyses of expression profiles of 494 rhythmically expressed mouse testicular lncRNAs in the six-time points desynchronized dataset under light dark (LD) condition. (A-E) Analysis of all the 494 rhythmically expressed mouse testicular lncRNAs: BioDare2 plot (A) of all the 494 rhythmically expressed lncRNAs, and 2D structures of the representative lncRNAs (B-E). (F-J) Analysis of 200 morning lncRNAs: BioDare2 plot (F) of all the 200 rhythmically expressed morning lncRNAs, and 2D structures of the representative lncRNAs (G-J). (K-O) Analysis of 191 evening lncRNAs: BioDare2 plot (K) of 191 rhythmically expressed evening lncRNAs, and 2D structures of the representative lncRNAs (L-O). (P-T) Analysis of 103 night lncRNAs: BioDare2 plot (P) of 103 rhythmically expressed evening lncRNAs, and 2D structures of the representative lncRNAs (Q-T).


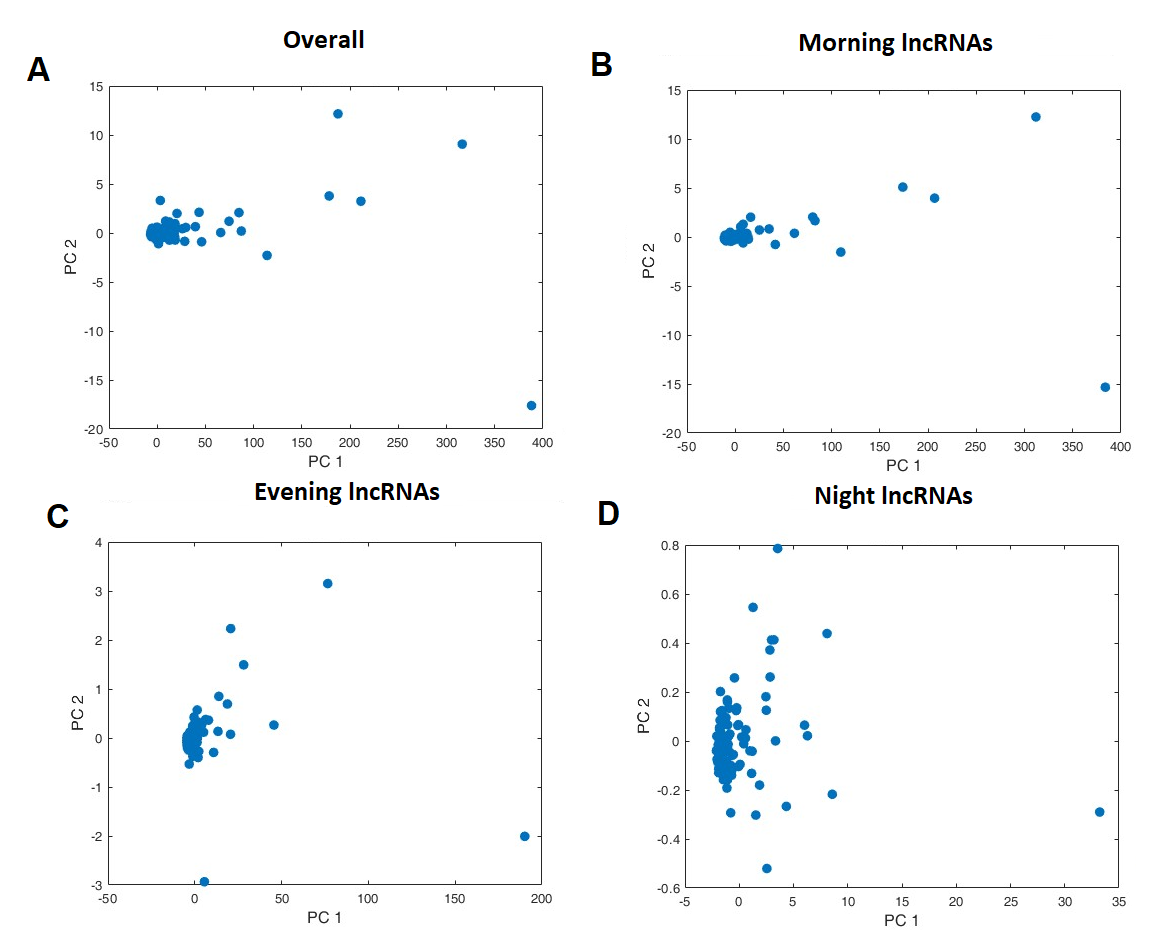


**Figure S14.** Principal Component Analysis (PCA) of 494 rhythmically expressed mouse testicular lncRNAs in the six-time-point desynchronized dataset under light dark (LD) condition. PCA plots of all the 494 rhythmically expressed lncRNAs (A), 200 morning lncRNAs (B), 191 evening lncRNAs (C), and 103 night lncRNAs (D).

**
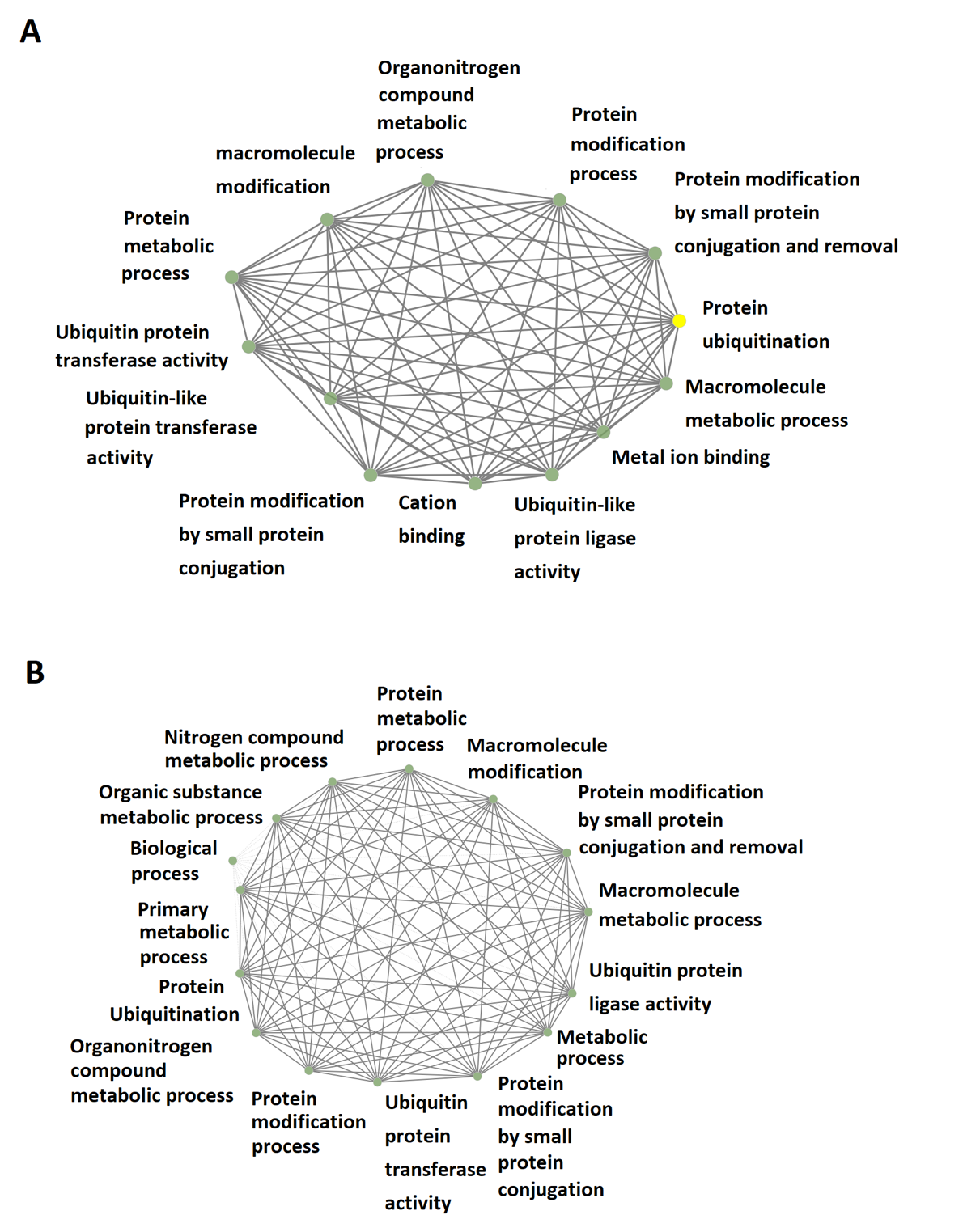
**

**Figure S15.** GO and KEGG analyses of rhythmically expressed mouse testicular lncRNAs in the six-time-point desynchronized dataset. (A) GO analysis of these lncRNAs revealed their possible involvement in numerous biological processes, such as, protein modification process, and cation binding. (B) KEGG enrichment analysis identified these lncRNAs’ potential biological functions, such as protein metabolic process, macromolecule modification, and protein ubiquitination.


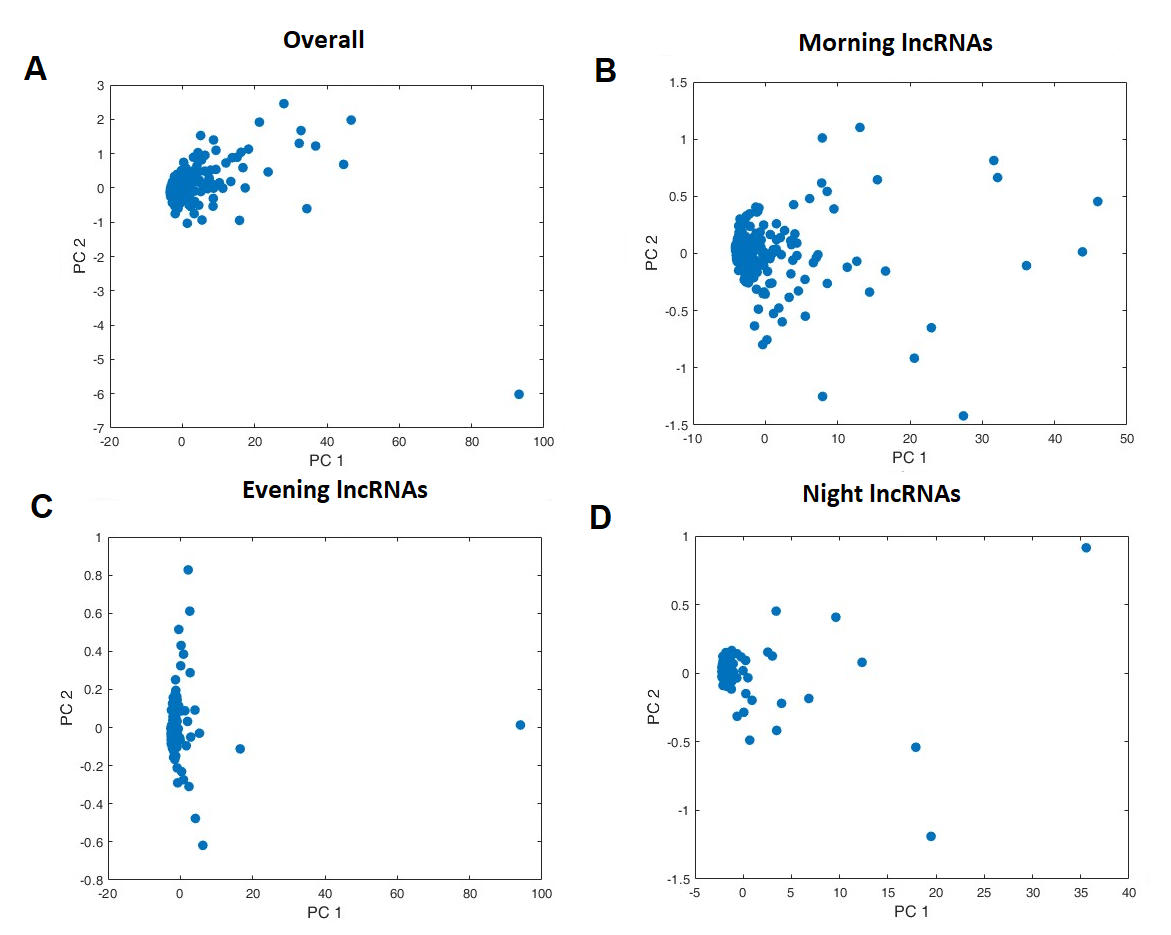


**Figure S16.** Principal Component Analysis (PCA) of 427 rhythmically expressed mouse testicular lncRNAs that lost rhythmicity in the desynchronized condition. PCA plots of all the 427 rhythmically expressed lncRNAs (A), 234 morning lncRNAs (B), 104 evening lncRNAs (C), and 89 night lncRNAs (D).


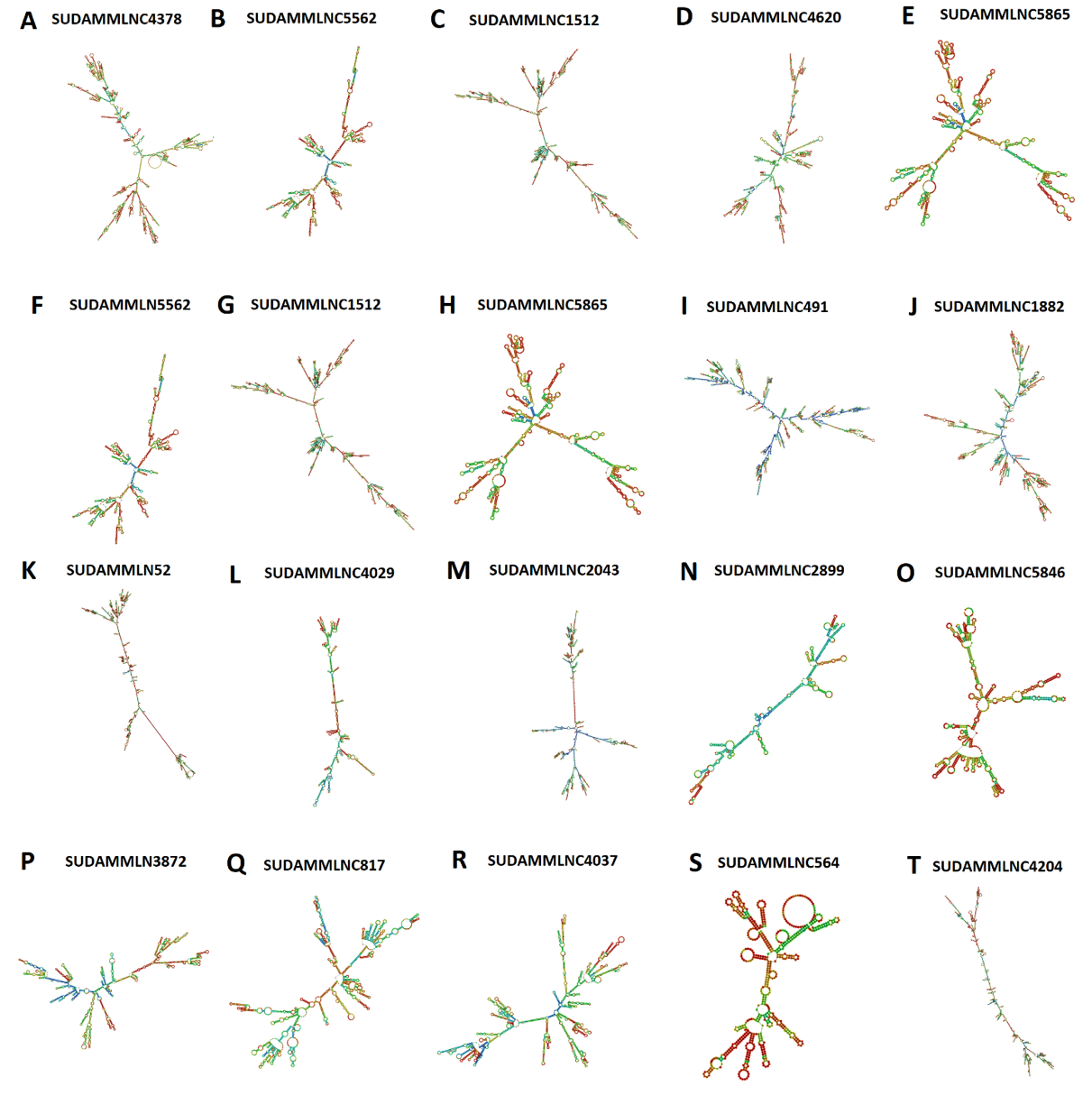


**Figure S17.** Computationally predicted secondary structures of the representative lncRNAs from the 427 lncRNAs that lost rhythmicity in the desynchronized condition with 6-point data from the control condition. (A-E) Secondary structures of the five representative lncRNAs from the overall 427 lncRNAs. (F-J) Secondary structures of the five representative lncRNAs from the 234 morning lncRNAs. (K-O) Secondary structures of the five representative lncRNAs from the 104 evening lncRNAs. (P-T) Secondary structures of the five representative lncRNAs from the 89 night lncRNAs.


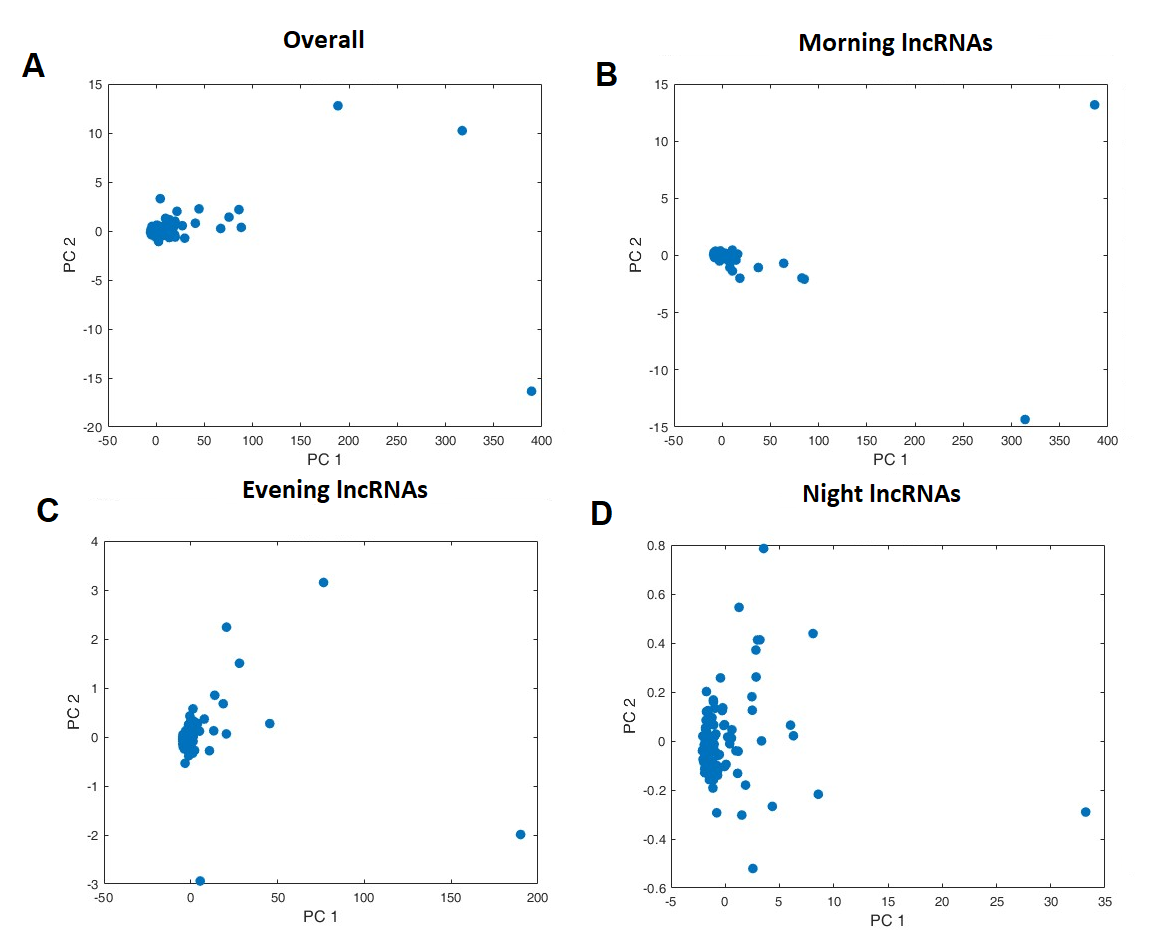


**Figure S18.** Principal Component Analysis (PCA) of 446 rhythmically expressed mouse testicular lncRNAs that gained rhythmicity in the desynchronized condition. PCA plots of all the 446 rhythmically expressed lncRNAs (A), 174 morning lncRNAs (B), 176 evening lncRNAs (C), and 96 night lncRNAs (D).


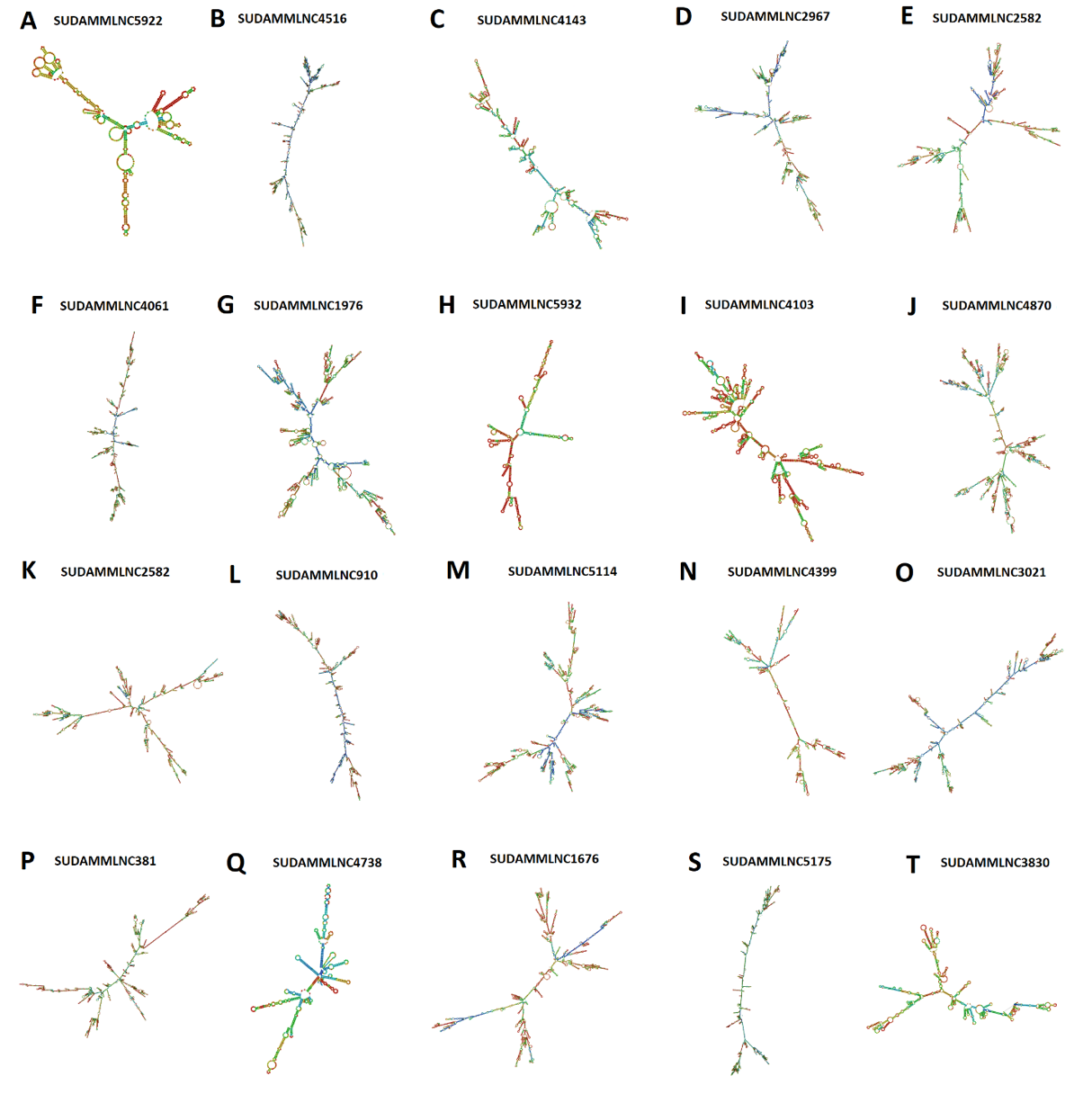


**Figure S19.** Computationally predicted secondary structures of the representative lncRNAs from the 446 lncRNAs that gained in the desynchronized condition. (A-E) Secondary structures of the five representative lncRNAs from the overall 446 lncRNAs. (F-J) Secondary structures of the five representative lncRNAs from the 174 morning lncRNAs. (K-O) Secondary structures of the five representative lncRNAs from the 176 evening lncRNAs. (P-T) Secondary structures of the five representative lncRNAs from the 96 night lncRNAs.


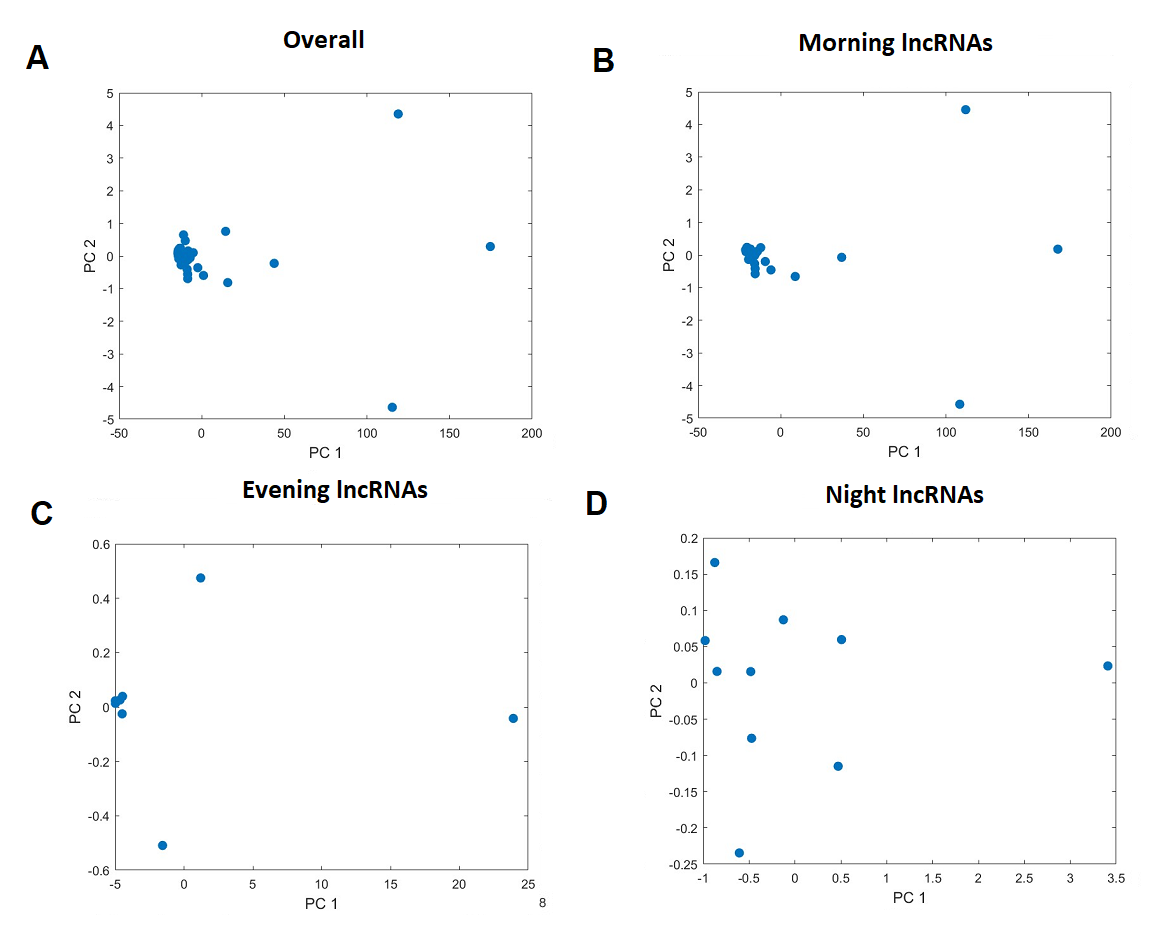


**Figure S20.** Principal Component Analysis (PCA) of the 48 rhythmicity-maintaining mouse testicular lncRNAs between the six-point control and desynchronized datasets with rhythmically expressed data from the six-time-point control dataset. PCA plots of all the 48 rhythmically expressed lncRNAs (A), 30 morning lncRNAs (B), eight evening lncRNAs (C), and 10 night lncRNAs (D).


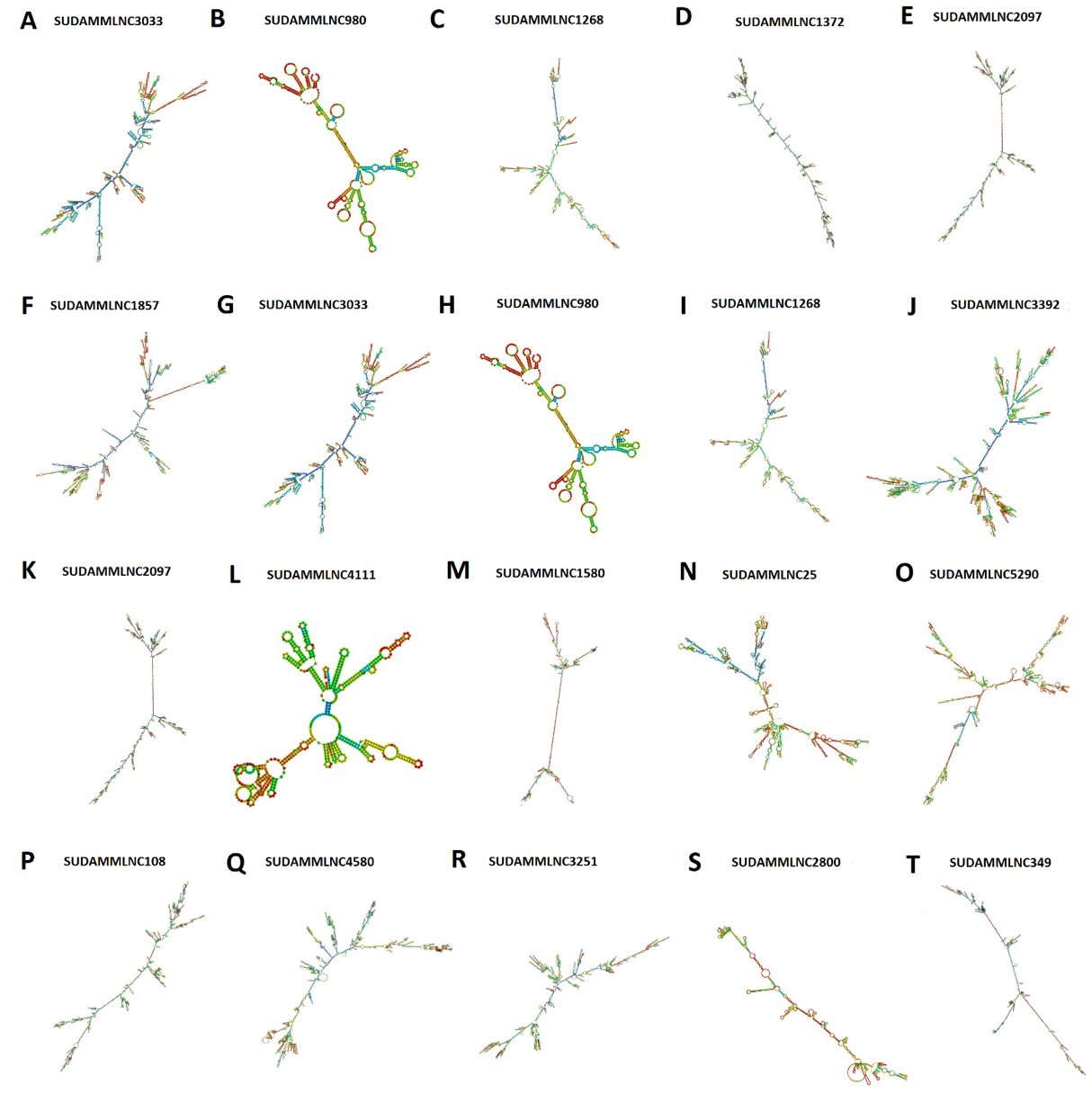


**Figure S21.** Computationally predicted secondary structures of the representative lncRNAs from the 48 rhythmicity-maintaining lncRNAs between the six-time-point control and desynchronized conditions. (A-E) Secondary structures of the five representative lncRNAs from the overall 48 lncRNAs. (F-J) Secondary structures of the five representative lncRNAs from the 30 morning lncRNAs. (K-O) Secondary structures of the five representative lncRNAs from the eight evening lncRNAs. (P-T) Secondary structures of the five representative lncRNAs from the 10 night lncRNAs.


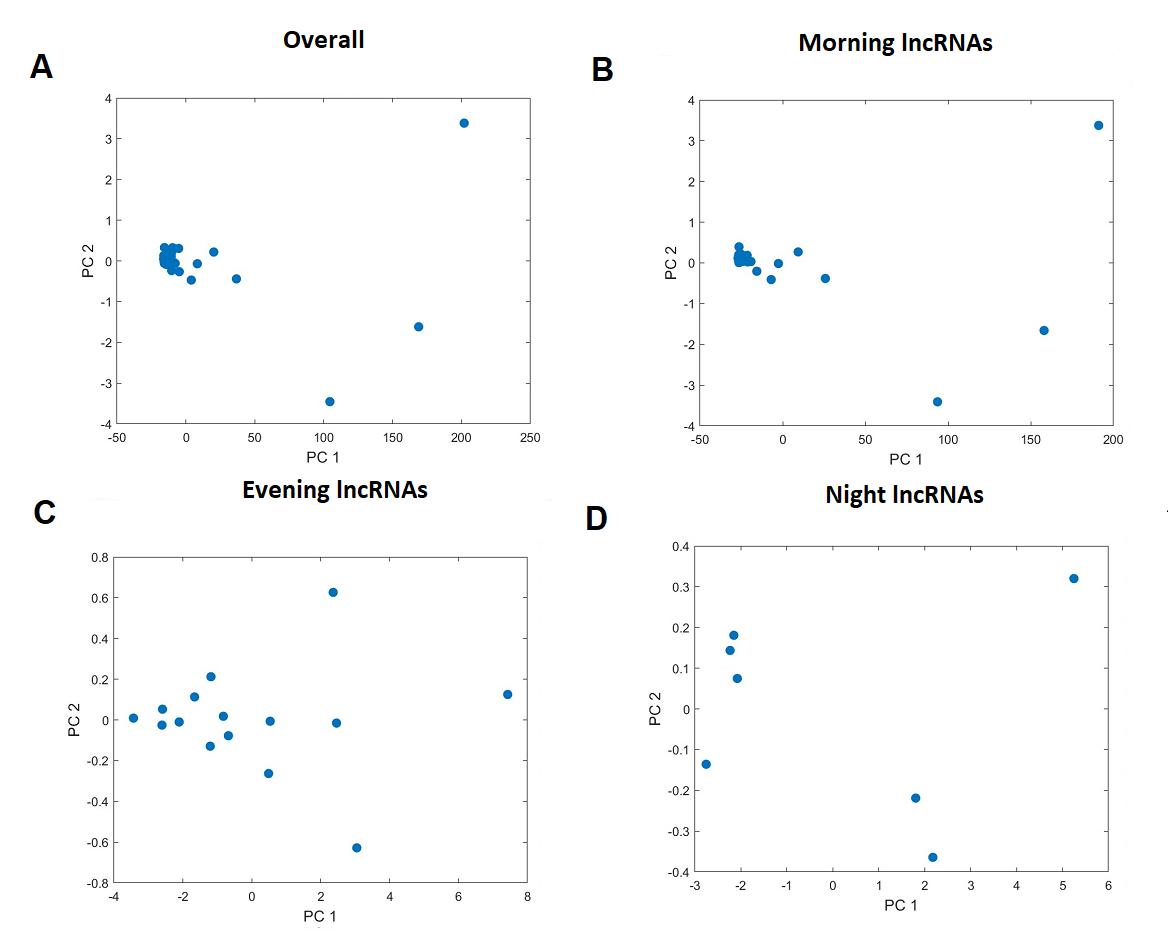


**Figure S22.** Principal Component Analysis (PCA) of the 48 rhythmicity-maintaining mouse testicular lncRNAs between the six-point control and desynchronized datasets with rhythmically expressed data from the six-time-point desynchronized dataset. PCA plots of the all 48 rhythmically expressed lncRNAs (A), 26 morning lncRNAs (B), 15 evening lncRNAs (C), and seven night lncRNAs (D).

**
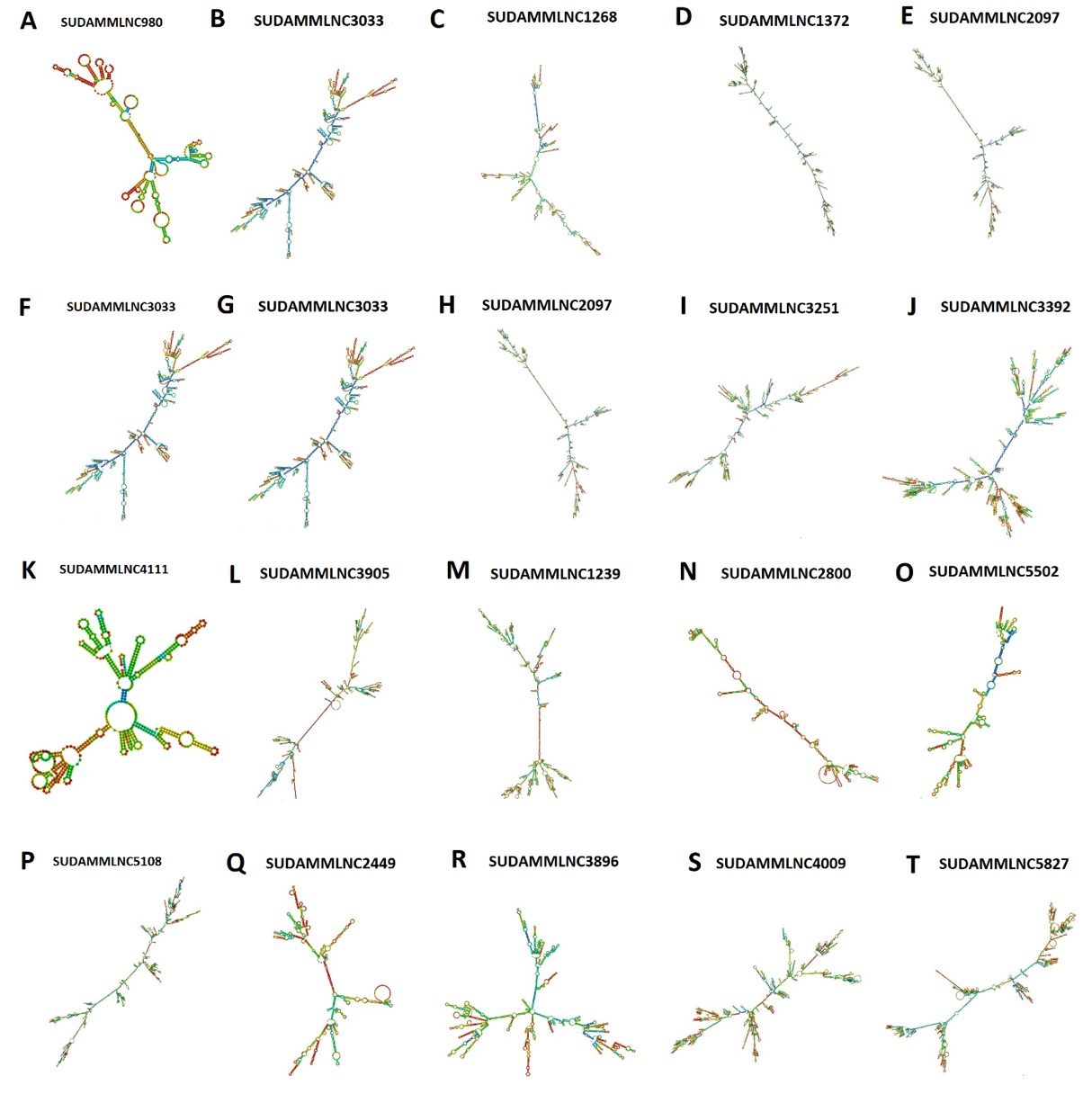
**

**Figure S23.** Computationally predicted secondary structures of the representative lncRNAs from 48 rhythmicity-maintaining mouse testicular lncRNAs between the six-point control and desynchronized datasets with 6-point data from the desynchronized condition. (A-E) Secondary structures of the five representative lncRNAs from the overall 48 lncRNAs. (F-J) Secondary structures of the five representative lncRNAs from the 26 morning lncRNAs. (K-O) Secondary structures of the five representative lncRNAs from the 15 evening lncRNAs. (P-T) Secondary structures of the five representative lncRNAs from the seven night lncRNAs.
